# Non-specific chemical inhibition of the Fanconi anemia pathway sensitizes cancer cells to cisplatin

**DOI:** 10.1186/1476-4598-11-26

**Published:** 2012-04-26

**Authors:** Céline Jacquemont, Julian A Simon, Alan D D'Andrea, Toshiyasu Taniguchi

**Affiliations:** 1 Howard Hughes Medical Institute, Chevy Chase, MD, USA; 2 Division of Human Biology, Fred Hutchinson Cancer Research Center, Seattle, WA, USA; 3 Division of Public Health Sciences, Fred Hutchinson Cancer Research Center, Seattle, WA, USA; 4 Division of Clinical Research, Fred Hutchinson Cancer Research Center, Seattle, WA, USA; 5 Department of Radiation Oncology, Dana-Farber Cancer Institute, Harvard Medical School, Boston, MA, USA

**Keywords:** Fanconi anemia, Drug resistance, Cisplatin, Small molecule, Homologous recombination

## Abstract

**Background:**

Platinum compounds such as cisplatin and carboplatin are DNA crosslinking agents widely used for cancer chemotherapy. However, the effectiveness of platinum compounds is often tempered by the acquisition of cellular drug resistance. Until now, no pharmacological approach has successfully overcome cisplatin resistance in cancer treatment. Since the Fanconi anemia (FA) pathway is a DNA damage response pathway required for cellular resistance to DNA interstrand crosslinking agents, identification of small molecules that inhibit the FA pathway may reveal classes of chemicals that sensitize cancer cells to cisplatin.

**Results:**

Through a cell-based screening assay of over 16,000 chemicals, we identified 26 small molecules that inhibit ionizing radiation and cisplatin-induced FANCD2 foci formation, a marker of FA pathway activity, in multiple human cell lines. Most of these small molecules also compromised ionizing radiation-induced RAD51 foci formation and homologous recombination repair, indicating that they are not selective toward the regulation of FANCD2. These compounds include known inhibitors of the proteasome, cathepsin B, lysosome, CHK1, HSP90, CDK and PKC, and several uncharacterized chemicals including a novel proteasome inhibitor (Chembridge compound 5929407).

Isobologram analyses demonstrated that half of the identified molecules sensitized ovarian cancer cells to cisplatin. Among them, 9 demonstrated increased efficiency toward FA pathway-proficient, cisplatin-resistant ovarian cancer cells. Six small molecules, including bortezomib (proteasome inhibitor), CA-074-Me (cathepsin B inhibitor) and 17-AAG (HSP90 inhibitor), synergized with cisplatin specifically in FA-proficient ovarian cancer cells (2008 + FANCF), but not in FA-deficient isogenic cells (2008). In addition, geldanamycin (HSP90 inhibitor) and two CHK1 inhibitors (UCN-01 and SB218078) exhibited a significantly stronger synergism with cisplatin in FA-proficient cells when compared to FA-deficient cells, suggesting a contribution of their FA pathway inhibitory activity to cisplatin sensitization.

**Conclusion:**

Our findings suggest that, despite their lack of specificity, pharmaceutical inhibition of the FA pathway by bortezomib, CA-074-Me, CHK1 inhibitors or HSP90 inhibitors may be a promising strategy to sensitize cisplatin-resistant, FA pathway-proficient tumor cells to cisplatin. In addition, we identified four new small molecules which synergize with cisplatin. Further development of their analogs and evaluation of their combination with cisplatin may lead to the development of efficient cancer treatments.

## Background

Platinum compounds, such as cisplatin (*cis*-diamminedichloroplatinum(II)) and carboplatin, are DNA interstrand crosslink (ICL)-inducing agents. ICLs bind both strands of the DNA helix, inhibit DNA replication and RNA transcription, and induce cell cycle arrest and apoptosis [1]. Platinum compounds are widely used for the treatment of multiple cancers, including ovarian, testicular, lung and some pediatric tumors [2]. Ovarian cancers initially respond very well to platinum-based therapy. However, many patients with ovarian cancer eventually relapse with platinum-resistant disease.

Various platinum resistance mechanisms have been proposed [2], including restoration of DNA repair [3]. Therefore, combination therapy using small molecules that inhibit DNA repair pathways responsible for cellular resistance to ICLs, such as Fanconi anemia (FA) pathway inhibitors, is a logical strategy to overcome and prevent platinum resistance.

FA is a rare genetic disease characterized by chromosomal instability, cancer susceptibility, aplastic anemia and cellular hypersensitivity to ICLs [4,5]. The 15 FA proteins [6,7] cooperate in the FA pathway, which coordinates multiple DNA repair mechanisms including endonuclease-mediated DNA processing, translesion DNA synthesis and homologous recombination (HR) [4,5]. Monoubiquitination and nuclear foci formation of FANCD2 and FANCI are crucial steps in the activation of this pathway [4,5,8]. The USP1/UAF1 deubiquitinase complex deubiquitinates FANCD2 and reverses the FA pathway activation [9]. Mutation and silencing of genes controlling the FA pathway have been linked to the development of tumors [10], and are associated with increased ICL sensitivity. Restoration of an intact FA pathway leads to the emergence of ICL-resistant tumors [10-12]. Thus, small molecules that inhibit the FA pathway may function as platinum chemo-sensitizers and have clinical utility in restoring platinum sensitivity of tumor cells.

We have developed a cell-based screening assay for small molecules that inhibit the FA pathway, and published partial results focusing on one of the hits, curcumin [13]. Monoketone analogs of curcumin were subsequently shown to have potent FA pathway inhibitory effects [14]. A cell-free screening assay using Xenopus egg extract also identified 2,3-dichloro-5,8-dihydroxy-1,4-naphthoquinone as an FA pathway inhibitor [15]. Recently, the Nedd8 activated enzyme (NAE) inhibitor MLN4924 was shown to sensitize cells to DNA damaging agents through indirect inhibition of the Fanconi anemia pathway [16]. However, despite important efforts, no specific inhibitor of the FA pathway has been identified so far.

In the current study, using a human cell-based assay, we completed screening of more than 16,000 chemicals for molecules that inhibit the FA pathway, and identified 26 small molecules that inhibit ionizing radiation (IR)-induced FANCD2 foci formation. We further characterized these compounds for their ability to inhibit RAD51 foci assembly, HR, or proteasome activity, and we compared their ability to sensitize ovarian cancer cells to cisplatin. We show that about half of these chemicals sensitized ovarian cancer cells to cisplatin, with in most cases a significantly stronger synergism in FA-proficient cells than in FA-deficient cells, suggesting that their effects are, at least partially, mediated through inhibition of the FA pathway.

## Results

### Cell-based screening for small molecules that inhibit the FA pathway

**Figure 1:**
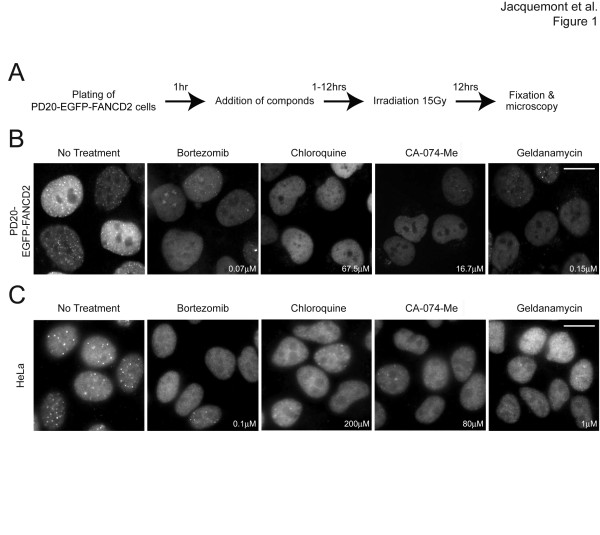
**Chemical library screening for small molecules that inhibit the Fanconi anemia pathway. (A)** Schematic of the screening for small molecules that inhibit IR-induced FANCD2 foci formation. **(B)** Representative photomicrographs of EGFP-FANCD2 foci in PD20F-EGFP-FANCD2 cells untreated and treated with the indicated compounds at the indicated concentration. The cells were treated with compounds immediately before irradiation (15 Gy), and fixed after 12 hours. **(C)** FANCD2 foci in HeLa cells untreated and treated with the indicated compounds at the indicated concentration. The cells were fixed 8 hours after irradiation (10 Gy) and immunostained with anti-FANCD2 antibody. Scale bar = 20 μm.

Assembly of DNA damage-induced FANCD2 foci is a widely used indicator of upstream FA pathway integrity [8]. To identify novel small molecules that inhibit the FA pathway, PD20-EGFP-FANCD2 cells [13] were treated with chemical libraries and exposed to IR (15 Gy) to induce FANCD2 foci formation. A significant decrease in the proportion of cells with IR-induced EGFP-FANCD2 foci upon drug treatment was scored as positive (Figure 1A). Using this cell-based assay, we tested more than 16,000 chemicals, and identified 43 compounds (0.27%) that significantly reduced EGFP-FANCD2 foci formation in the initial screen ( Additional file 1: Table S1), including curcumin, wortmannin, alsterpaullone and H-9, as previously described [13]. Fifteen of these 43 compounds were then confirmed to inhibit IR-induced FANCD2 foci formation in multiple cell lines, including PD20-FANCD2, U2OS, HeLa and TOV21G + FANCF ovarian cancer cells, using a wide range of drug concentrations (Table 1, Figures1B and 1C, Additional file 2: Figure S1, and data not shown). Interestingly, some of the drugs independently identified through this screen shared common inhibitory features (Table 1): curcumin [17] and compound 5929407 (see below) are proteasome inhibitors, and curcumin, H-9, and Gö6976 are PKC inhibitors.

**Table 1:** List of the validated small molecule inhibitors of the FA pathway

**Chemicals**	**Primary known functions**	**IC50 (μM)**
Lactacystin	Proteasome inhibitor	1.7
MG132	Proteasome and calpain inhibitor	0.8
ALLN	Proteasome, calpain, cathepsin L inhibitor	9.2
5929407	Proteasome inhibitor (this study)	6.5
Curcumin	Proteasome, protein kinase C (PKC), EGF-receptor tyrosine kinase, IkappaB kinase and mTOR inhibitor	18.6
H-9	PKA, PKC, PKG inhibitor	34.2
Gö6976	PKC, CHK1 inhibitor	> 50
SB218078	CHK1, Cdc2, PKC inhibitor	1.4
UCN-01	PKC, CHK1, CDK, AKT inhibitor	0.14
Alsterpaullone	CDK, GSK3 inhibitor	1.6
Roscovitine	CDK, ERK1,2 inhibitor	27.5
Geldanamycin	HSP90 inhibitor	0.15
17-AAG	HSP90 inhibitor	0.6
CA-074-Me	CathepsinB inhibitor	16.7
Chloroquine	Lysosome permeabilisation, inhibition of drug efflux pumps (ATP-binding cassette transporters)	67.5
Wortmannin	Casein kinase II, phosphatidylinositol 3-kinase (PI 3-kinase), polo-like kinase 1 (PLK1) inhibitor	84.4
DRB	Casein kinase II, RNA pol II inhibitor	11
HNMPA-(AM)3	Insulin receptor tyrosine kinase inhibitor	4.7
Puromycin	Protein synthesis inhibitor	5.2
TPEN	Heavy metal chelator	5
5656325	Unknown	0.83
5315179	Unknown	5.9
7012246	Unknown	7
5195243	Unknown	2.2
5373662	Unknown	1.3

The identified chemicals are presented with their major known functions and the dose required to inhibit 50% of IR-induced EGFP-FANCD2 foci formation (IC50) in PD20-EGFP-FANCD2 cells. Chemical structures of 5195243 (2-nitro-9,10-phenanthrenedione 10-oxime), 5373662 (2-(2-pyrinylmethylene)quinuclidin-3-one), 5656325 (1-(2-methoxy-1-naphtyl)-3-(3-pyridinyl)-2-propen-1-one), 5929407 (5-[4-(dietylamino)benzylidene]-3-{[(3-methylphenyl)amino]methyl}-2-thioxo-1,3-thiazolidin-4-one), 5315179 (1-(2,4-dimethoxyphenyl)-3-(3-pyridinyl)-2-propen-1-one) and 7012246 (1-(5-bromo-2-hydroxyphenyl)-3-(3-pyridinyl)-2-propen-1-one)) are shown in Additional file 2: Figure S1.

Eleven additional compounds, related to the chemicals identified in our primary screen or identified in unrelated studies [18], were also subjected to secondary screening: two CHK1/PKC inhibitors (UCN-01, SB218078), a CDK inhibitor (roscovitine), an HSP90 inhibitor (17-AAG), four proteasome inhibitors (bortezomib, MG132, ALLN, lactacystin), two compounds structurally related to 5656325 (5315179 and 7012246 ( Additional file 2: Figure S1)), and chloroquine. All of these compounds inhibited DNA damage-induced FANCD2 foci assembly in multiple cell lines, without altering the overall expression of EGFP-FANCD2 or endogenous FANCD2 (Figure 1B Additional file 3: Figure S5 and data not shown). The dose required to inhibit 50% of IR-induced EGFP-FANCD2 foci formation (IC_50_) in PD20-EGFP-FANCD2 cells was determined for each of these 26 compounds (Table 1). Importantly, 18 of them exhibited IC_50_ values lower than 10 μM. Although the FA pathway inhibition capacity of these inhibitors may not be due to specific targeting of components of the FA pathway, we will refer to them as FA pathway inhibitors in the remaining text for simplicity.

### Identification of a novel proteasome inhibitor among the small molecules that inhibit the FA pathway

**Figure 2:**
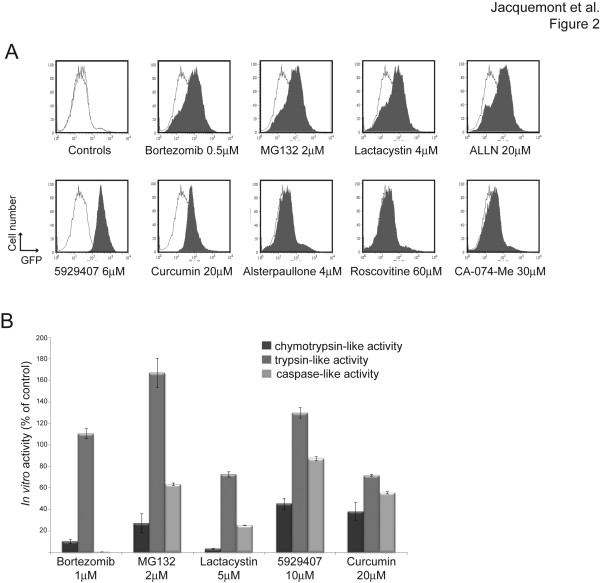
**Identification of compound 5929407 as a proteasome inhibitor. **(A) *In vivo* proteasome activity assay. Flow cytometry diagrams showing proteasome inhibition-dependent expression of GFP in GFPu-1 cells treated with the indicated drugs (gray area), compared with non-treated cells (white area). In the first panel, three independent non-treated samples are plotted to show variability. (B) Compounds with proteasome inhibitor activity preferentially inhibit proteasome chymotrypsin- and caspase-like activities. Proteasome activities were monitored using fluorogenic probes processed by cellular extracts obtained from HeLa cells exposed to the various proteasome inhibitors (n = 3, mean ± SEM).

All proteasome inhibitors tested (bortezomib, MG132, ALLN, lactacystin, curcumin) inhibited FANCD2 foci formation in multiple cell lines (Table 1 and [18]). Therefore, we hypothesized that some of the newly identified FA pathway inhibitors could also inhibit the proteasome. We first tested proteasome activity using GFPu-1 cells, in which inhibition of proteasome results in increased GFP expression [19]. All proteasome inhibitors as well as the Chembridge compound 5929407 induced a strong increase in GFP expression in GFPu-1 cells (Figure 2A).

We then assessed the effects of these compounds on the three proteases activities associated with the proteasome (trypsin-like, chymotrypsin-like and caspase-like activities), using fluorogenic compounds in HeLa cells extracts. All compounds that increased GFP expression in GFPu-1 cells (bortezomib, MG132, lactacystin, ALLN, curcumin and 5929407) inhibited chymotrypsin- and caspase-like activities of the proteasome, the chymotrypsin-like activity being generally the most affected. In addition, lactacystin and curcumin inhibited trypsin-like activity (Figure 2B). These findings indicate that the compound 5929407 is a novel proteasome inhibitor that preferentially inhibits the chymotrypsin-like activity of the proteasome.

### Most chemicals that inhibit the FA pathway inhibit homologous recombination

Since the FA pathway is required for efficient DNA double-strand break repair by HR [4,20], we tested whether the identified compounds affect HR efficiency in human cells using the DR-GFP reporter system [21]. In this assay, GFP expression reflects the occurrence of an HR repair event; compounds that disrupt HR repair will decrease GFP expression. Twenty-four hours after transfection of a HA-tagged I-Sce1-encoding plasmid, U2OS-DR-GFP cells were exposed to the identified FA pathway inhibitors for 24 hours ( Additional file 4A and B: Figures S2A and B). The concentration used for each drug was optimized to induce minimal decrease in cell viability (more than 90% viable cells after 24 hours, Additional file 4C: figure S2C), and did not significantly affect HA-tagged I-Sce1 expression (averaged 36.3% of the population for all drugs in multiple experiments compared to 39.2% in the non-treated population), with the exception of MG132, UCN-01, compounds 5195423 and 7012246 ( Additional file 4D: Figure S2D). The HA-positive population was analyzed for GFP expression. In the absence of drug, 9.5 ± 0.9% of the HA-positive cells expressed GFP.

**Figure 3:**
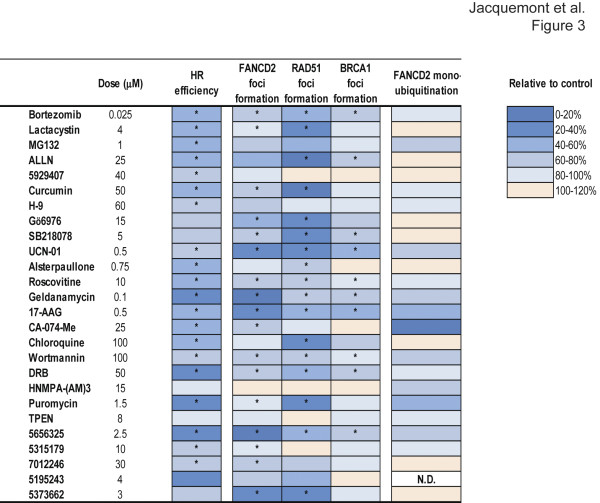
**Effects of 26 chemicals that inhibit the FA pathway on HR efficiency, IR-induced foci formation of FANCD2, RAD51 and BRCA1, and IR-induced FANCD2 monoubiquitination.** (See Additional file 4: Figures S2, Additional file 6: Figure S4, and Additional file 3: Figure S5 for details) Color-coded representation of HR efficiency in DR-GFP assay, the proportion of cells with IR-induced foci of the indicated proteins, and the proportion of FANCD2 monoubiquitinated form on Western blot (8 hours after 10 Gy), compared to untreated controls. U2OS-DR-GFP cells were used for all the experiments. An asterisk (*) indicates significant decrease compared to controls (p ≤0.05, paired *t* test, n = 3 to 7) in DR-GFP and foci formation experiments. N.D. = not determined.

With the exception of SB218078, HNMPA-(AM)_3_ and TPEN, all FA pathway inhibitors significantly decreased HR (p ≤0.05) (Figure 3 and Additional file 4E and F: Figures S2E and S2F). No significant differences in the cell cycle distribution were observed under these conditions, except for UCN-01, 17-AAG, wortmannin, HNMPA-(AM)_3_ and compounds 5929407 and 5315179 ( Additional file 5A: Figure S3A).

BRCA1 and RAD51 are required for efficient HR and are known to interact with FANCD2 [8,22-24]. We therefore tested whether the FA pathway inhibitors block FANCD2, BRCA1 and RAD51 foci formation upon DNA damage in U2OS-DR-GFP cells. To do so, we used drug treatments identical to those used during the DR-GFP assay, consisting in longer exposure (24 hours) to lower concentrations of chemicals than the initial screen and confirmation experiments. Under such conditions, most of the drugs still significantly inhibited IR-induced foci formation of FANCD2 and RAD51 (Figure 3 and Additional file 6: Figure S4), without significantly modifying cell cycle distribution ( Additional file 5B: Figure S3B). The drugs that failed to significantly inhibit FANCD2 foci formation under these conditions demonstrated significant inhibition at higher dosage (data not shown), consistently with the initial screen. IR-induced monoubiquitination of FANCD2 was in most cases moderately inhibited or unaffected, with the exception of CA-074-Me, which strongly inhibited it ( Additional file 3: Figure S5). IR-induced foci formation of BRCA1 was also mildly affected or unaffected by the compounds (Figure 3 and Additional file 6: Figure S4). By using higher concentrations for a shorter time (8 hours drug exposure), we observed that most drugs significantly inhibited IR-induced FANCD2, RAD51 and BRCA1 foci formation, as well as IR-induced FANCD2 monoubiquitination, in the absence of significant variations of cell cycle distribution (data not shown). In addition, most of the drugs significantly inhibited cisplatin-induced FANCD2 foci formation in 24 hours co-treatment experiments ( Additional file 7: Figure S6). These results demonstrate that most FA pathway inhibitors inhibit HR processes (RAD51 foci formation and HR repair) in addition to FANCD2 foci formation, indicating that the identified chemicals target multiple steps of the DNA damage response pathway and are not specific for FA pathway inhibition. The lack of inhibition of FANCD2 monoubiquitination suggests that the FA pathway inhibitors may inhibit processes involved in the recruitment of proteins at sites of damage, rather than damage signaling upstream of FANCD2 monoubiquitination.

### Identification of the compounds that synergize with cisplatin in ovarian cancer cells

Since the integrity of the FA pathway is critical for cellular resistance to ICL-inducing agents such as cisplatin, FA pathway inhibitors may sensitize tumor cells to cisplatin in an FA pathway-dependent manner. To test this hypothesis, we performed isobologram analyses on the ovarian cancer cell line 2008 [25], which is deficient in the FA pathway due to hypermethylation of the *FANCF* promoter, and on its isogenic, complemented FA pathway-proficient counterpart 2008 + FANCF cell line [11].

First, single agent survival curves were generated, and the dose producing a 50% reduction of cell survival (LD50, lethal dose 50%) was determined ( Additional file 8: Table S2). As previously reported [11], 2008 cells were significantly more sensitive to cisplatin than 2008 + FANCF cells. 2008 and 2008 + FANCF cells were equally sensitive to all FA pathway inhibitors, except for puromycin and geldanamycin. Higher tolerance of 2008 + FANCF cells to puromycin was likely due to the use of puromycin selection to generate the complemented cell line, and therefore, puromycin was omitted from further analysis. The reason for the differential sensitivity of 2008 and 2008 + FANCF cells toward geldanamycin remains unknown.

Next, isobolograms at the LD50 level were generated following the method previously described [26]. Survival assays were performed using the combination of 10 cisplatin concentrations with 6 concentrations of each FA pathway inhibitor. LD50/LD50_0_ unit (drug concentration necessary to induce 50% death when combined with cisplatin divided by the drug’s LD50 in the absence of cisplatin) values of each FA pathway inhibitor were plotted against corresponding LD50/LD50_0_ unit values of cisplatin. The distribution of points along the line connecting values of 1 corresponds to an additive effect of the two drugs while scattering below or above represents synergism and antagonism, respectively. In addition, combination index (CI) values were calculated according to Chou and Tallay’s method [26]; CI ≤ 0.90 indicates synergism.

**Figure 4:**
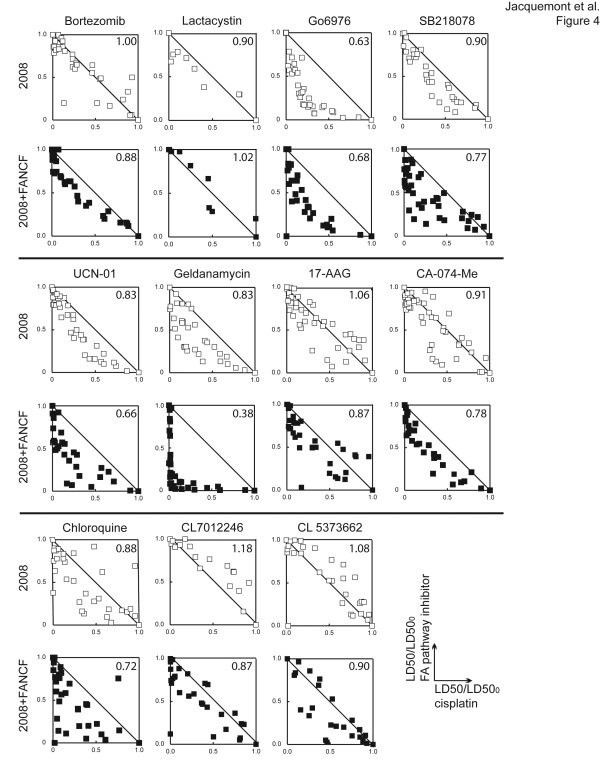
**Eleven chemicals that inhibit the FA pathway synergized with cisplatin in the killing of ovarian cancer cells.** Isobolograms at the LD50 level obtained in at least 3 independent experiments performed with FA pathway-deficient ovarian cancer cells (2008) and their FA pathway-proficient counterpart (2008 + FANCF) are presented (except of lactacystin, for which the result of only one experiment is shown, because of the high concentration required to achieve 50% killing). Average combination index (CI) is indicated in the upper right corner of each isobologram: a CI ≤ 0.90 indicates synergism. Results are summarized in Table 2

Analyses performed at 50% killing revealed that 11 FA pathway inhibitors exhibited synergism with cisplatin in 2008 and/or 2008 + FANCF cells (Figure 4, Additional file 9: Figure S7, summarized in Table 2). Bortezomib, 17-AAG, CA-074-Me, and compounds 7012246 and 5373662 synergized with cisplatin in FA pathway-proficient 2008 + FANCF cells, but not in their isogenic FA pathway-deficient counterpart 2008, consistent with their FA pathway inhibition activity. Geldanamycin, three CHK1 inhibitors (Gö6976, UCN-01, SB218078) and chloroquine synergized with cisplatin in both 2008 and 2008 + FANCF cells. Geldanamycin, UCN-01 and SB218078 exhibited a significantly stronger synergism in 2008 + FANCF cells than in 2008 cells (p ≤ 0.01, unpaired *t* test), again consistent with their FA pathway inhibition activity. Finally, lactacystin weakly synergized with cisplatin in 2008 cells only. The other compounds had either additive or antagonistic effect with cisplatin.

**Table 2:** Drug interactions at 50% killing between cisplatin and the chemicals that inhibit the FA pathway in FA pathway-deficient/proficient ovarian cancer cells

	**Interaction with cisplatin**			
	**2008**		**2008 + FANCF**	
**Chemicals**	***FA-deficient***		***FA-proficient***	
	**CI**	**Interpretation**	**CI**	**Interpretation**
Bortezomib	1.00 ± 0.05	Additive	0.88 ± 0.02	**Moderate synergism**
Lactacystin	0.90 ± 0.05	**Slight synergism**	1.02 ± 0.05	Additive
MG132	1.13 ± 0.04	*Slight antagonism*	1.13 ± 0.05	*Slight antagonism*
ALLN	1.17 ± 0.04	*Slight antagonism*	1.06 ± 0.09	Additive
5929407	1.10 ± 0.04	Additive	1.00 ± 0.04	Additive
Curcumin	0.98 ± 0.02	Additive	1.06 ± 0.02	Additive
H-9	1.08 ± 0.04	Additive	0.98 ± 0.04	Additive
Gö6976	0.63 ± 0.04	**Synergism**	0.68 ± 0.03	**Synergism**
SB218078	0.90 ± 0.05	**Slight synergism**	0.77 ± 0.03	**Moderate synergism**
UCN-01	0.83 ± 0.03	**Moderate synergism**	0.66 ± 0.04	**Synergism**
Alsterpaullone	1.05 ± 0.03	Additive	1.15 ± 0.03	*Slight antagonism*
Roscovitine	0.98 ± 0.03	Additive	0.98 ± 0.03	Additive
Geldanamycin	0.83 ± 0.04	**Moderate synergism**	0.38 ± 0.05	**Synergism**
17-AAG	1.06 ± 0.10	Additive	0.87 ± 0.04	**Slight synergism**
CA-074-Me	0.91 ± 0.03	Additive	0.78 ± 0.02	**Moderate synergism**
Chloroquine	0.88 ± 0.05	**Slight synergism**	0.72 ± 0.05	**Moderate synergism**
Wortmannin	1.08 ± 0.05	Additive	1.12 ± 0.05	*Slight antagonism*
DRB	1.08 ± 0.04	Additive	0.97 ± 0.05	Additive
HNMPA-(AM)3	0.96 ± 0.02	Additive	0.91 ± 0.03	Additive
Puromycin	1.08 ± 0.03	Additive	0.88 ± 0.04	**Slight synergism**
TPEN	1.16 ± 0.08	*Slight antagonism*	1.13 ± 0.04	*Slight antagonism*
5656325	0.99 ± 0.03	Additive	1.05 ± 0.05	*Slight antagonism*
5315179	1.14 ± 0.09	*Slight antagonism*	0.94 ± 0.04	Additive
7012246	1.18 ± 0.05	*Slight antagonism*	0.87 ± 0.04	**Slight synergism**
5195243	1.22 ± 0.04	*Moderate antagonism*	1.02 ± 0.02	Additive
5373662	1.08 ± 0.04	Additive	0.90 ± 0.04	**Slight synergism**

Combination index (CI) at 50% killing values (mean ± SEM) calculated from isobologram at the LD50 level analyses of combination of cisplatin with each FA pathway inhibitor, performed in FA-deficient (2008) and FA-proficient (2008 + FANCF) ovarian cancer cell lines. See Figure 4 and Additional file 9: Figure S7 for details. Interpretation of combination index values: 0.10 < CI ≤ 0.30, strong synergism; 0.30 < CI ≤ 0.70, synergism; 0.70 < CI ≤ 0.85, moderate synergism; 0.85 < CI ≤ 0.90, slight synergism; 0.90 < CI ≤ 1.10, additivity; 1.10 < CI ≤ 1.20, slight antagonism; 1.20 < CI ≤ 1.45, moderate antagonism [26]. Synergism is highlighted in bold letters.

Analyses performed at 70% killing (Additional file 10: Table S3) showed that bortezomib, 17-AAG, and CA-074-Me, in addition to geldanamycin, Gö6976, and UCN-01, synergized with cisplatin in both 2008 and 2008 + FANCF cells. ALLN, SB218078, and compounds 5656325, 5315179 and 5373662 synergized with cisplatin in 2008 + FANCF only.

Taken together, about half of the FA pathway inhibitors sensitized ovarian cancer cells to cisplatin (11 of 25 at 50% killing (Table 2) and 12 of 25 at 70% killing ( Additional file 10: Table S3)). Most of them exhibit a significantly stronger synergism with cisplatin in FA pathway-proficient 2008 + FANCF cells than in 2008 cells (CI significantly lower for 2008 + FANCF than 2008 (p ≤ 0.05) for 8 of 11 drugs at 50% killing, for 9 of 12 drugs at 70% killing), indicating that their effects are, at least partially, mediated through inhibition of the FA pathway.

We also examined whether the compounds that most significantly synergized with cisplatin would sensitize cells to IR ( Additional file 11: Figure S8 and Additional file 12: Table S4). Geldanamycin and SB218078 synergized with IR in both 2008 and 2008 + FANCF cells, Gö6976 in 2008, and compound 5373662 in 2008 + FANCF cells. Other compounds showed additive effects with IR.

## Discussion

Here we identified 26 chemicals that inhibit the formation of IR- and cisplatin-induced FANCD2 foci. Many demonstrated a stronger inhibition of FANCD2 foci formation than FANCD2 mono-ubiquitination, suggesting that at lower concentrations they interfere with FANCD2 recruitment at site of DNA damage more than with FANCD2 mono-ubiquination. However, the cathepsinB inhibitor CA-074-Me demonstrated a stronger inhibition of FANCD2 mono-ubiquitination than foci formation, suggesting the intriguing possibility that recruitment of FANCD2 at sites of DNA damages may be supported with reduced levels of mono-ubiquitinated FANCD2. Additionally, most chemicals also inhibited IR-induced RAD51 foci formation and DNA double-strand break repair by HR, but generally not BRCA1 foci formation, indicating that they inhibit multiple discrete steps of the DNA damage response and are not specific inhibitors of the Fanconi anemia pathway.

Many of the identified chemicals appeared to cluster around common targets, such as the proteasome, PKC, CHK1, CDK, HSP90, cathepsin B and lysosome function, or casein kinase II (Table 1). Some of these targets have already been implicated in the FA pathway and HR. For instance, proteasome function is required for activation of the FA pathway and HR [18,27]. Consistent with this, among the new FA pathway inhibitors, we identified a novel and uncharacterized proteasome inhibitor (5929407). ATR and its downstream kinase, CHK1, which can be directly or indirectly inhibited by UCN-01, Gö6976, SB218078, alsterpaullone, roscovitine and wortmannin, are involved in FA pathway activation [28-31]. CHK1 inhibition also inhibits RAD51 binding to DNA [32]. HSP90 is also implicated in the FA pathway and HR, since FANCA, BRCA2, CHK1 and CDKs are clients of HSP90 [33-35]. CDK inhibition leads to perturbation of cell cycle, proliferation and checkpoints, and compromises CHK1, BRCA2 and RAD51 functions [36,37], which can lead to impaired FA pathway and HR. A possible role for PKC, cathepsin B, lysosome and casein kinase II in the regulation of the FA pathway and HR has not been reported yet, and is worth testing in the future. Whether these chemicals directly target some components of the FA pathway remains to be determined. Further studies of the pathways affected by these inhibitors may shed light on new regulatory mechanisms of the FA pathway and HR.

A total of 14 out of the 26 chemicals that inhibit the FA pathway sensitized ovarian cancer cells to cisplatin (Table 2 and Additional file 10: Table S3). The majority showed a stronger synergism with cisplatin in FA-proficient than in FA-deficient cells, suggesting that FA pathway inhibitory activity of these compounds contributes to the cisplatin sensitization. The chemicals that synergized with cisplatin in both FA pathway-deficient and -proficient cells probably did so through mechanisms independent of the FA pathway, such as inhibition of RAD51 recruitment and HR, or other mechanisms. The inhibition of the FA pathway and these other mechanisms may independently or synergistically participate in the increased sensitization to cisplatin observed using these chemicals. Most synergistic interactions between FA pathway inhibitors and cisplatin were stronger at higher killing levels, suggesting that these combinations are relevant for cancer therapy.

Although the role of the FA pathway in cellular resistance to ICL-inducing agents, such as cisplatin, has been established, some FA pathway inhibitors did not synergize with cisplatin. Their activity on targets other than the FA pathway may prevent chemosensitization. Alternatively, cisplatin treatment may alleviate their toxicity. It is also possible that the effects of combining cisplatin and the inhibitors vary in cell type- and context-specific manners. Whether the inhibitors synergize with cisplatin in different types of tumor cells remains to be systematically determined.

CHK1 inhibitors have been used in preclinical and clinical trials to treat p53-deficient and, more recently, p53-proficient cancers [38-41]. A CHK1 inhibitor, Gö6976, has been suggested to sensitize FA-deficient cells to cisplatin [42]. Our results showed that CHK1 inhibitors sensitized p53 wild-type, FA-proficient and -deficient ovarian cancer cells to cisplatin. SB218078 and UCN-01 showed a significantly stronger synergism with cisplatin in the FA-proficient cell line than in the FA-deficient cell line (p = 0.01 and 2x10^-5^ respectively at 50% killing), while no difference between the two cell lines was detected with Gö6976.

HSP90 inhibitors have also been shown to sensitize tumor cells to DNA damaging agents including cisplatin [43,44]. In the current study, geldanamycin and, to a lesser extent, 17-AAG sensitized cells to cisplatin. Downregulation of multiple HSP90 clients involved in the FA pathway and HR (FANCA, BRCA2, CHK1, CDK) may result in the observed sensitization to cisplatin. However, a recent phase I clinical trial in patients with refractory tumors for combination therapy using cisplatin and 17-AAG demonstrated that the combination had anti-tumor activity, but exhibited significant toxicity, preventing any phase II development [45].

Chemicals that inhibit proteasome (bortezomib and, more marginally, lactacystin and ALLN) or lysosome function (CA-074-Me and chloroquine) sensitized ovarian cancer cells to cisplatin. Bortezomib and CA-074-Me showed a stronger synergism with cisplatin in FA-proficient than in FA-deficient cells, consistent with inhibition of the FA pathway by these drugs. The mechanism of FA pathway inhibition by these chemicals remains unknown. Proteasomes and lysosomes are protein degradation systems that can contribute to cellular tolerance to various proteotoxic stressors, and can confer resistance to chemo-, radio- and immunotherapy [46]. It is possible that perturbed protein degradation interferes with the FA pathway. Alternatively, the FA pathway may require activity of these protein degradation machineries. Chloroquine has already demonstrated potential to enhance the effect of radiation therapy and chemotherapy with vincristine, Akt inhibitors, and histone deacetylase inhibitors through its inhibition of lysosome function and autophagy [47]. Our study suggests that chloroquine can potentiate the cytotoxic effects of cisplatin. Combination of chloroquine and cisplatin is undergoing a clinical trial for the treatment of small cell lung cancer (http://clinicaltrials.gov/). This work suggests that combination of chloroquine and cisplatin may also have therapeutic advantages in cisplatin-resistant ovarian cancer treatment. Combinations of bortezomib and platinum compounds (cisplatin or carboplatin) are also undergoing clinical trials for the treatment of ovarian and other cancers (http://clinicaltrials.gov/).

Our study identified four Chembridge compounds without known bioactivities as FA pathway inhibitors that can sensitize ovarian cancer cells to cisplatin. Three of these compounds have a related structure (5656325, 5315179 and 7012246), and show some synergism with cisplatin at higher killing level ( Additional file 10: Table S3). Interestingly, compound 5373662 showed synergism with cisplatin and with IR in FA-proficient cells only. Further analyses of its mechanism(s) of action, as well as analyses of related compounds, are warranted.

The ATM kinase, involved in DNA damage response, has been identified as a synthetic lethal gene in FA-deficient cells [48]. Whether the FA pathway inhibitors specifically kill ATM-deficient tumor cells is another important question.

In summary, this study underscores the potential clinical benefit of combination therapy using cisplatin and inhibitors of CHK1, HSP90, and protein degradation machineries (proteasome and lysosome), during treatment of cisplatin-resistant tumors. In addition, we identified four new small molecules that synergize with cisplatin (and, in one case, IR). Our results provide a rationale for further development of new generations of analog drugs with improved specificity and decreased toxicity, as well as pre-clinical testing in appropriate animal models. Further evaluation of these combinations in cisplatin-resistant tumors may lead to the development of efficient cancer treatments.

## Materials and methods

### Cell lines and culture conditions

HeLa, U2OS, TOV-21 G and GFPu-1 cells were purchased from the American Type Culture Collections (Manassas, VA). 2008 and FANCF-corrected 2008 + FANCF ovarian cancer cells, TOV-21 G and FANCF-corrected TOV-21 G + FANCF ovarian cancer cells were described previously [11]. FANCD2-deficient fibroblast line (PD20), PD20 corrected with wild-type FANCD2 (PD20-FANCD2) and enhanced green fluorescent protein (EGFP)-FANCD2 (PD20-EGFP-FANCD2 clone 7) were described previously [13]. U2OS-DR-GFP cells were a gift from Drs. Maria Jasin and Koji Nakanishi [49]. Cell lines were grown in DMEM supplemented with 10% fetal calf serum. Gamma irradiation was delivered using a JL Shepherd Mark I Cesium Irradiator (JL Shepherd & Associates).

The present research has been approved by the Institutional Review Board (IRB) Committee at the Fred Hutchinson Cancer Research Center (IRB Protocol file number 6023).

### Chemicals

The chemical libraries (ICCB bioactives (489 compounds), Commercial Diversity Set 1 (5,056 compounds), Chembridge DiverSet Library (10,000 compounds) and NINDS II library (1,040 compounds) were used to identify inhibitors of the FA pathway. For subsequent studies, chemicals were purchased from Biomol (lactacystin, penitrem A, splitomicin, (±)-13-HODE), EMD biochemicals (actinomycin D, AG213, AG370, ALLN, alsterpaullone, α-amanitin, BAPTA-AM, bumetanide, CA-074-Me, curcumin, DRB, geldanamycin, Gö6976, HNMPA-(AM)_3_, H-9, K-252c, MG132, nifedipine, propidium iodide, puromycin, roscovitine, SB218078, spermine NONOate, TPEN, trichostatin A, wortmannin), Cayman Chemical (leukotriene B_3_), Chembridge Corporation (5195243, 5315179, 5373662, 5656325, 5929407, 7012246), Fisher (chloroquine), Millenium Pharmaceutical (bortezomib), MP (3-methyladenine), Sigma (cisplatin, UCN-01), Tocris (ochratoxin A), VWR (17-AAG).

### Screen for small molecules that inhibit the FA pathway

The PD20-EGFP-FANCD2 clone 7 was used in the screen [13]. The cell-based screening of ICCB bioactives and Commercial Diversity Set 1 was done at the Institute for Chemistry and Cell Biology (Boston, MA) and a partial result was previously reported [13]. The cell-based screening of Chembridge DiverSet Library and NINDS II library was done at Fred Hutchinson Cancer Research Center. For this screening, duplicate 96-well plates were seeded with PD20-EGFP-FANCD2 clone 7 cells (10,000 cells per well). Chemical compounds from the library were added, five compounds per well, at a single concentration of 7.5 μmol/L. After a 12-hour incubation, cells were irradiated (15 Gy) and fixed for EGFP microscopy 12 hours later. Photomicrographs were obtained for each well and wells with significant (50%) reduction in percentage of EGFP-FANCD2 foci positive cells were identified by visual inspection. The five compounds of each well identified with reduced EGFP-FANCD2 foci were then individually tested.

**Immunofluorescence microscopy** was performed as described previously [18]. Antibodies against BRCA1 (D-9, Santa Cruz, 1/100), γH2AX (JBW301, Upstate, 1/1000), FANCD2 (NB 100–182, Novus Biologicals, 1/1000) and RAD51 (PC130, Calbiochem, 1/1000 or H-92, Santa Cruz, 1/200) were used. Species-specific fluorescein isothiocyanate (FITC)- or Cy3-conjugated secondary antibodies (Jackson Immunoresearch (West Grove, PA)) diluted in blocking buffer (1/2000) were incubated for 1 hour at room temperature. Images were acquired using a microscope (TE2000, Nikon, Tokyo, Japan) equipped with a 40x immersion objective (1.3NA) and a CCD camera (CoolSnap ES, Photometrics, Tucson, AZ) controlled by MetaVue (Universal Imaging, Downington, PA) and analyzed using MetaVue or ImageJ softwares. At least 100 cells per experimental point were scored for presence of foci, and each experiment repeated at least 3 times independently.

### Flow cytometric analyses

Exponentially growing cells were plated in drug-free medium 48 hours before experiment. For proteasome activity assay [19], GFPu-1 cells were exposed to drugs at the indicated concentration for 24 hours, then analyzed for green fluorescent protein (GFP) expression. For cell cycle analyses, cells were exposed to drugs at the indicated concentration for 24 hours, and exposed to IR 8 hours before being pulse-labeled with 30 μM 5-Bromo-2’-deoxy-Uridine (BrdU (Sigma)) for 15 minutes, washed and fixed with 70% ice-cold ethanol. Cells were then stained for DNA content (propidium iodide, PI) and BrdU incorporation with anti-BrdU rat monoclonal antibody (MAS250, Harlan Sera Lab, UK) followed by FITC-conjugated goat anti-rat antibody (Jackson Immunoresearch). For HR assays, cells were transfected with pCBASce (a hemagglutinin (HA)-tagged I-Sce1 expression vector) [50] or the empty pCAGGS vector using TransIT transfection reagent (Mirus) following manufacturer recommendations. 24 hours after transfection, cells were treated with the indicated drugs at the indicated concentration for 24 hours. Cells were then fixed and stained for HA expression with mouse anti-HA antibody (HA.11, Convance, USA, 1/1000) followed by APC-conjugated donkey anti-mouse antibody (Jackson Immunoreserach). To specifically determine the proportion of HR events in I-Sce1 expressing cells, the percentage of GFP-positive cells among the HA-positive cell population was quantified. Flow cytometric analyses were performed on a Becton Dickinson FACScan. Fluorescence data were plotted using FlowJo (Tree Star, Inc., Ashland, OR). At least three independent experiments were carried out for each condition.

**Proteasome activity fluorogenic assays** were performed as in [51]. Briefly, HeLa cells were treated with the indicated FA pathway inhibitors for 6 hours, scrapped, washed in cold PBS, and lysed by 30 minutes incubation in 5 mM EDTA on ice. Cellular extracts were cleared by centrifugation (10,000 rpm, 5 min, 4°C) and quantified. Fluorogenic peptides specific for the chymotrypsin-like, trypsin-like and caspase-like activities of the proteasome (Suc-LLVY-AMC, Boc-LLR-AMC, Z-LLQ-AMC respectively (Bachem)) were incubated with 5 ug HeLa extracts in specific substrate buffers [51]. Fluorescence emitted by proteasome cleavage of the peptides was monitored every 200 seconds for 1 hour using a fluorometer (Hitachi F-4500 fluorometer) with 380 nm and 440 nm excitation and emission filters, respectively, and maximum linear slopes were measured. Emission of serial dilutions of AMC in extracts was used for fluorometer calibration. Proteasome activity was calculated as concentration of AMC (pM) produced per second per mg of protein. Three independent experiments were performed.

### Drug interaction analysis

2008 and 2008 + FANCF cells were plated in 96-well plates at a density of 2000 cells/well. 24 h after plating, cisplatin and FA pathway inhibitors were added concomitantly, or FA pathway inhibitors were added and the cells immediately exposed to IR. Cytotoxicity was measured using the standard crystal violet assay 6 days after drug addition: cells were washed twice with PBS, fixed for 5 minutes at room temperature in 10% (v/v) methanol and 10% (v/v) acetic acid. Adherent colonies were stained for 2 to 10 minutes with 1% (w/v) crystal violet (Sigma) in methanol, rinsed in distilled water, and dried before the adsorbed dye was re-solubilized with methanol containing 0.1% (w/v) SDS by gentle agitation for 1 to 4 hours at room temperature. Dye concentration was quantified using ELx800 Universal Microplate Reader (Bio-Tek Instruments, Inc.) at 595 nm. For quantitation, readings of absorbance at 595 nm were normalized to those obtained from untreated cells, assumed to yield 100% cell survival, and empty wells, considered to be 0% cell survival. Cytotoxicity results were analyzed as described [52]. Briefly, after each experiment, survival curves were generated, for cisplatin and each FA pathway inhibitor alone and for the drug combinations. The LD50s for each drug in combination were determined, and LD50/LD50_0_ units were derived as ratio of LD50 for cisplatin or IR and the FA pathway inhibitor relative to LD50 of each drug alone (this value was designated as 1) for each cell line. Isobolograms were generated at LD50 levels. Each plot presents values generated in at least three independent experiments. In addition, combination index (CI) values were calculated by the use of the Chou and Talladay method [26]. An identical analysis was performed at the 70% killing level.

**Western blot analysis** was done as described [18]. Anti- FANCD2 (NB 100–182, Novus Biologicals, 1/20,000 dilution) and HRP-conjugated ECL anti-rabbit IgG (Amersham, 1/5000) were used. Films were digitalized using a standard scanner and images processed using ImageJ.

## Abbreviations

APC: Allophycocyanine; BrdU: 5-Bromo-2’-deoxy-Uridine; CDK: Cyclin-dependant kinase; CHK1: Checkpoint kinase 1; DAPI: 4’, 6-diamidino-2-phenylindole dihydrochloride; EGFP: Enhanced green fluorescent protein; FA: Fanconi anemia; FITC: Fluorescein isothiocyanate; GFP: Green fluorescent protein; HA: Hemagglutinin; HR: Homologous recombination; HSP90: Heat shock protein 90; ICL: Interstrand crosslink; IR: Ionizing radiation; LD50: Lethal dose 50%; PKC: Protein kinase C.

## Competing interests

The authors declare that they have no conflict of interest.

## Authors’ contributions

CJ and TT carried out the experimental work and data analysis, CJ, ADA and TT designed the study, JS provided critical reagents, and CJ and TT wrote the manuscript, all authors edited and approved the manuscript.

## Supplementary Material

Additional file 1 **Table S1.** List of the chemicals scored as positives in the primary screening. The 43 compounds scored as FA pathway inhibitors in the primary screening are listed. The 15 compounds that wre verified in the secondary screenings are indicated with “X”. Click here for file

Additional file 2 **Figure S1.** Chemical structure of the Chembridge library compounds that inhibit DNA damage-induced FANCD2 foci formation. Compounds 5195243, 5373662, 5929407 and 5656325 were identified by the compound library screening. Compounds 5315179 and 7012246 were later selected upon their 2D analogy with 5656325. Click here for file

Additional file 3 **Figure S5.** Some FA pathway inhibitors inhibit IR-induced FANCD2 monoubiquitination in U2OS-DR-GFP cells. U2OS-DR-GFP cells were exposed to FA pathway inhibitors for 24 hours using the concentrations used for the HR and foci formation assays, and irradiated with 10Gy 8 hours before the end of the exposure to the drugs. Whole cell lysates were subjected to FANCD2 western blotting. The ratio of ubiquitinated versus non- ubiquitinated FANCD2 is indicated for each condition. These results are summarized in Figure 3. Click here for file

Additional file 4 **Figure S2.** Most FA pathway inhibitors inhibit HR. (**A**) Schematic of the HR assay performed using FA pathway inhibitors. (**B**) Flow cytometric analysis for HR efficiency using U2OS-DR-GFP cells and HA-tagged I-Sce1. Cells fixed and stained with anti-HA and APC-linked specific secondary antibodies were first gated in FCS/SSC scatter, then HA-positive population was gated in an APC/GFP dot plot and analyzed for GFP expression using a red/GFP dot plot. (**C**) The relative proportion of live cells compared to non-treated control cells in the HR assay in U2OS-DRGFP cells are shown (mean ± SEM (n=3 to 7)). Percentage of live cells was 95.0±1.2% in non-treated condition. (**D**) The relative proportion of cells expressing HA-tagged ISce1 compared to non-treated control cells in the HR assay in U2OS-DRGFP cells are shown (mean ± SEM (n=3 to 7)). An asterix (*) indicates significant decrease in proportion of HA-positive cells upon exposure to the indicated drug (p≤0.05, t test). Percentage of HA-positive cells was 39.2±2.5% in non-treated condition. (**E**) The relative proportion of GFP-positive cells in the HA-positive population, compared to non-treated control cells, is shown (mean ± SEM (n=3 to 7)). An asterix (*) indicates significant decrease in the proportion of GFP-positive/HA-positive cells upon exposure to the indicated drug (p≤0.05, t test). Percentage of GFP-positive/HA-positive cells was 9.5±0.9% in non-treated condition. These results are summarized in Figure 3. (**F**) Examples of flow cytometric profiles obtained with U2OS-DR-GFP cells untreated and treated with geldanamycin (0.1μM) for 24 hours. Click here for file

Additional file 5 **Figure S3.** Most FA pathway inhibitors do not significantly affect the proportion of cells in S+G2phases of the cell cycle. Cell cycle distribution quantified using BrdU/PI staining of U2OS-DR-GFP cells treated for 24 hours with the indicated drugs (**A**), or treated for 24 hours with the indicated drugs and irradiated with 10Gy 8 hours before the end of treatment (**B**) (n=3 to 4). An asterix (*) indicates significant decrease in proportion of S+G2 cells upon exposure to the indicated drug (p≤0.05, t test). Examples of flow cytometric profiles obtained with non-treated and FA pathway inhibitor-treated cells are shown. Click here for file

Additional file 6 **Figure S4.Figure S2.** Most FA pathway inhibitors inhibit HR. (**A**) Schematic of the HR assay performed using FA pathway inhibitors. (**B**) Flow cytometric analysis for HR efficiency using U2OS-DR-GFP cells and HA-tagged I-Sce1. Cells fixed and stained with anti-HA and APC-linked specific secondary antibodies were first gated in FCS/SSC scatter, then HA-positive population was gated in an APC/GFP dot plot and analyzed for GFP expression using a red/GFP dot plot. (**C**) The relative proportion of live cells compared to non-treated control cells in the HR assay in U2OS-DRGFP cells are shown (mean ± SEM (n=3 to 7)). Percentage of live cells was 95.0±1.2% in non-treated condition. (**D**) The relative proportion of cells expressing HA-tagged ISce1 compared to non-treated control cells in the HR assay in U2OS-DRGFP cells are shown (mean ± SEM (n=3 to 7)). An asterix (*) indicates significant decrease in proportion of HA-positive cells upon exposure to the indicated drug (p≤0.05, t test). Percentage of HA-positive cells was 39.2±2.5% in non-treated condition. (**E**) The relative proportion of GFP-positive cells in the HA-positive population, compared to non-treated control cells, is shown (mean ± SEM (n=3 to 7)). An asterix (*) indicates significant decrease in the proportion of GFP-positive/HA-positive cells upon exposure to the indicated drug (p≤0.05, t test). Percentage of GFP-positive/HA-positive cells was 9.5±0.9% in non-treated condition. These results are summarized in Figure 3. (**F**) Examples of flow cytometric profiles obtained with U2OS-DR-GFP cells untreated and treated with geldanamycin (0.1μM) for 24 hours. Click here for file

Additional file 7 **Figure S6.** Most FA pathway inhibitors significantly inhibit cisplatin-induced foci formation of FANCD2 in U2OS-DR-GFP cells. Relative proportion of U2OS-DR-GFP cells with more than 10 foci after 24hour incubation with 5μM cisplatin and the indicated drug, compared to controls (mean ± SEM (n=3)). An asterisk (*) indicates significant decrease in the proportion of foci-positive cells compared to controls (p ≤0.05, t test). In non-treated conditions, the percentage of FANCD2-positive cells was 83.13 ± 8.3%. Examples of immunofluorescence staining are also shown. Scale bar = 20μm. N.D. = not determined. Click here for file

Additional file 8 **Table S2.** 2008 and 2008+FANCF cells are equally sensitive to most FA pathway inhibitors. Lethal dose 50 (LD50) (mean ± SEM (n=3 to 6)) of the FA pathway inhibitors in 2008 and 2008+FANCF cells. An asterisk (*) indicates significant difference in sensitivity between 2008 and 2008+FANCF cells (p ≤0.05, paired t test). N.D. = not determined. Only two experiments were performed using lactacystin, because of the high concentration required to achieve 50% killing. Click here for file

Additional file 9 **Figure S7.** Isobologram analyses at 50% killing of the FA pathway inhibitors that did not sensitize 2008 or 2008+FANCF ovarian cancer cells to cisplatin. Isobolograms at the LD50 level are presented, showing the results obtained in at least 3 independent experiments. Results are summarized in Table 2. Click here for file

Additional file 10 **Table S3.** Drug interactions at 70% killing between cisplatin and the FA pathway inhibitors in FA pathway-deficient and -proficient ovarian cancer cells. Combination index (CI) at 70% killing values (mean ± SEM) calculated from isobologram at the LD70 level analyses of combination of cisplatin with all FA pathway inhibitor, performed in an FA-deficient (2008) and an FA-proficient (2008+FANCF) ovarian cancer cell lines. Synergism is indicated in bold text. Click here for file

Additional file 11 **Figure S8.** Isobologram analyses for sensitization to IR of the FA pathway inhibitors that most significantly sensitize 2008 and/or 2008+FANCF ovarian cancer cells to cisplatin. Isobolograms at the LD50 level are presented, showing the results obtained in at least 3 independent experiments. Average combination index (CI) is indicated in the upper right corner of each isobologram: a CI≤0.90 indicates synergism. Results are summarized in Additional file 12: Table S4. Click here for file

Additional file 12 **Table S4.** Interactions at 50% killing between IR and the FA pathway inhibitors in FA pathway-deficient and -proficient ovarian cancer cells. Combination index (CI) at 50% killing values (mean ± SEM) calculated from isobologram at the LD50 level analyses of combination of IR with each FA pathway inhibitor, performed in FA-deficient (2008) and FA-proficient (2008+FANCF) ovarian cancer cell lines (see Additional file 11: Figure S8). Synergism is indicated in bold text. Click here for file
