# Supplementary material for: Non-specific chemical inhibition of the Fanconi anemia pathway sensitizes cancer cells to cisplatin
Source: Mol Cancer. 2012 Apr 26;11:26. doi: 10.1186/1476-4598-11-26 (PMC3478989; doi:10.1186/1476-4598-11-26)
Supplement: Additional file 1 — Table S1. List of the chemicals scored as positives in the primary screening. The 43 compounds scored as FA pathway inhibitors in the primary screening are listed. The 15 compounds that wre verified in the secondary screenings are indicated with “X”. [file 1476-4598-11-26-S1.docx]

| **Library** | **Chemicals** | **Primary known function** | **Validated in secondary screenings** |
| --- | --- | --- | --- |
| **ICCB bioactives (489 compounds)** | **27** |  | **11** |
|  |  |  |  |
|  | (+-)13-HODE | Racemic monohydroxy fatty acid resulting from the non-enzymatic oxidation of linoleic acid |  |
|  | 3-Methyladenine | Autophagy inhibitor |  |
|  | α-amanitin | RNA pol II inhibitor |  |
|  | Actinomycin D | Transcription inhibitor |  |
|  | AG213 | EGFR inhibitor |  |
|  | AG-370 | PDGFR kinase inhibitor |  |
|  | Alsterpaullone | CDK, GSK3 inhibitor | **X** |
|  | BAPTA-AM | Cell permeable Ca2+ chelator |  |
|  | Bumetamide | Na+ K+ CL+ cotransport inhibitor |  |
|  | CA-074-Me | CathepsinB inhibitor | **X** |
|  | Curcumin | Proteasome, protein kinase C (PKC), EGF-receptor tyrosine kinase, IkappaB kinase and mTOR inhibitor | **X** |
|  | DRB | Casein kinase II inhibitor, RNA pol II inhibitor | **X** |
|  | Geldanamycin | HSP90 inhibitor | **X** |
|  | Gö6976 | PKC, CHK1 inhibitor | **X** |
|  | H-9 | PKA, PKC, PKG inhibitor | **X** |
|  | HNMPA-(AM)3 | Insulin Receptor Tyrosine Kinase inhibitor | **X** |
|  | K252c | PKC inhibitor |  |
|  | Leukotriene B3 | LTA hydrolase metabolite of LTA_3_ in the leukotriene biosynthetic pathway, has pro-inflammatory effects |  |
|  | Nifedipine | Calcium-channel blocker |  |
|  | Ochratoxin | Stimulates endoplasmic reticulum Ca2+ ATPase |  |
|  | Penitrem A | Potassium channels inhibitor |  |
|  | Propidium iodide | DNA intercalator |  |
|  | Puromycin | Protein synthesis inhibitor | **X** |
|  | Spermine NONOate | NO donor |  |
|  | TPEN | Heavy metal chelator | **X** |
|  | Trichostatin-A | Histone deacetylases inhibitor |  |
|  | Wortmannin | Casein kinase II, phosphatidylinositol 3-kinase (PI 3-kinase), polo-like kinase 1 (PLK1) inhibitor | **X** |
| **Commercial Diversity Set 1 (5,056 compounds)** | **3** |  | **0** |
|  | PD00600 | Unknown |  |
|  | 5323069 | Unknown |  |
|  | 1M556S | Unknown |  |
| **Chembridge DiverSet (10,000 compounds)** | **12** |  | **4** |
|  | 5185048 | Unknown |  |
|  | 5194582 | Unknown |  |
|  | 5195243 | Unknown | **X** |
|  | 5233097 | Unknown |  |
|  | 5373662 | Unknown | **X** |
|  | 5429881 | Unknown |  |
|  | 5656325 | Unknown | **X** |
|  | 5929407 | Proteasome inhibitor (this study) | **X** |
|  | 5972795 | Unknown |  |
|  | 6037040 | Unknown |  |
|  | 6126765 | Unknown |  |
|  | 6143632 | Unknown |  |
| **NINDS II (1,040 compounds)** | **1** |  | **0** |
|  | 6102279 | Unknown |  |
|  |  |  |  |

**Table S1. List of the chemicals scored as positives in the primary screening.**

The 43 compounds scored as FA pathway inhibitors in the primary screening are listed. The 15 compounds that wre verified in the secondary screenings are indicated with “X”.
