# Supplementary figures and images for: Non-specific chemical inhibition of the Fanconi anemia pathway sensitizes cancer cells to cisplatin
Source: Mol Cancer. 2012 Apr 26;11:26. doi: 10.1186/1476-4598-11-26 (PMC3478989; doi:10.1186/1476-4598-11-26)

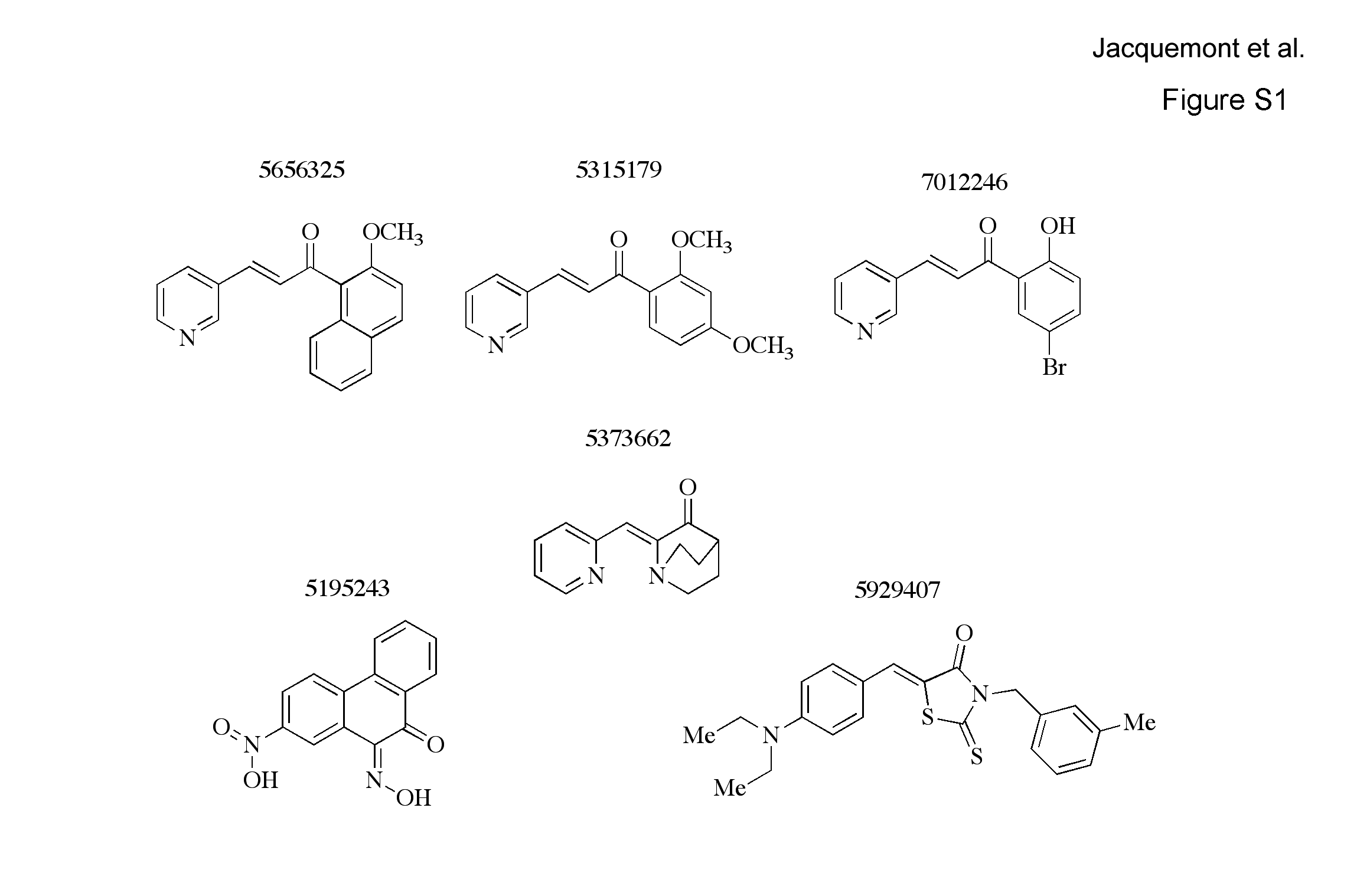

Supplement: Additional file 2 — Figure S1. Chemical structure of the Chembridge library compounds that inhibit DNA damage-induced FANCD2 foci formation. Compounds 5195243, 5373662, 5929407 and 5656325 were identified by the compound library screening. Compounds 5315179 and 7012246 were later selected upon their 2D analogy with 5656325. [file 1476-4598-11-26-S2.tiff]

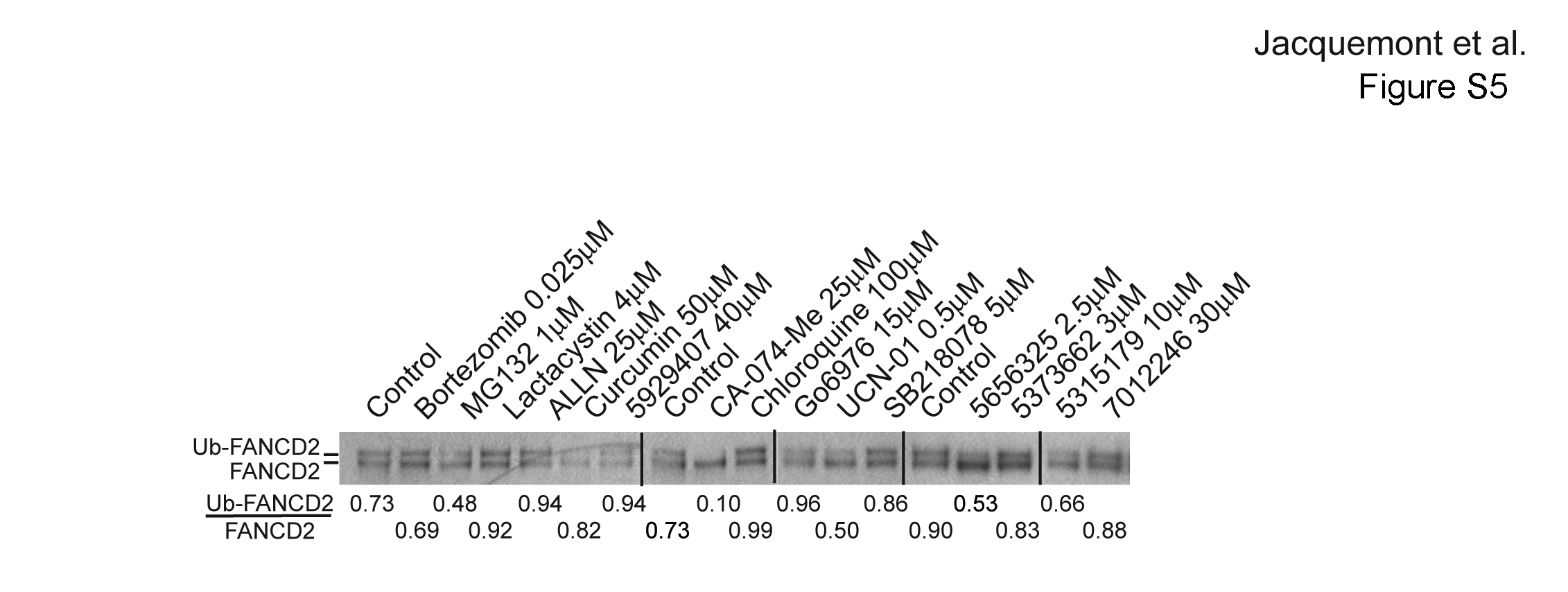

Supplement: Additional file 3 — Figure S5. Some FA pathway inhibitors inhibit IR-induced FANCD2 monoubiquitination in U2OS-DR-GFP cells. U2OS-DR-GFP cells were exposed to FA pathway inhibitors for 24 hours using the concentrations used for the HR and foci formation assays, and irradiated with 10Gy 8 hours before the end of the exposure to the drugs. Whole cell lysates were subjected to FANCD2 western blotting. The ratio of ubiquitinated versus non- ubiquitinated FANCD2 is indicated for each condition. These results are summarized in Figure 3. [file 1476-4598-11-26-S3.tiff]

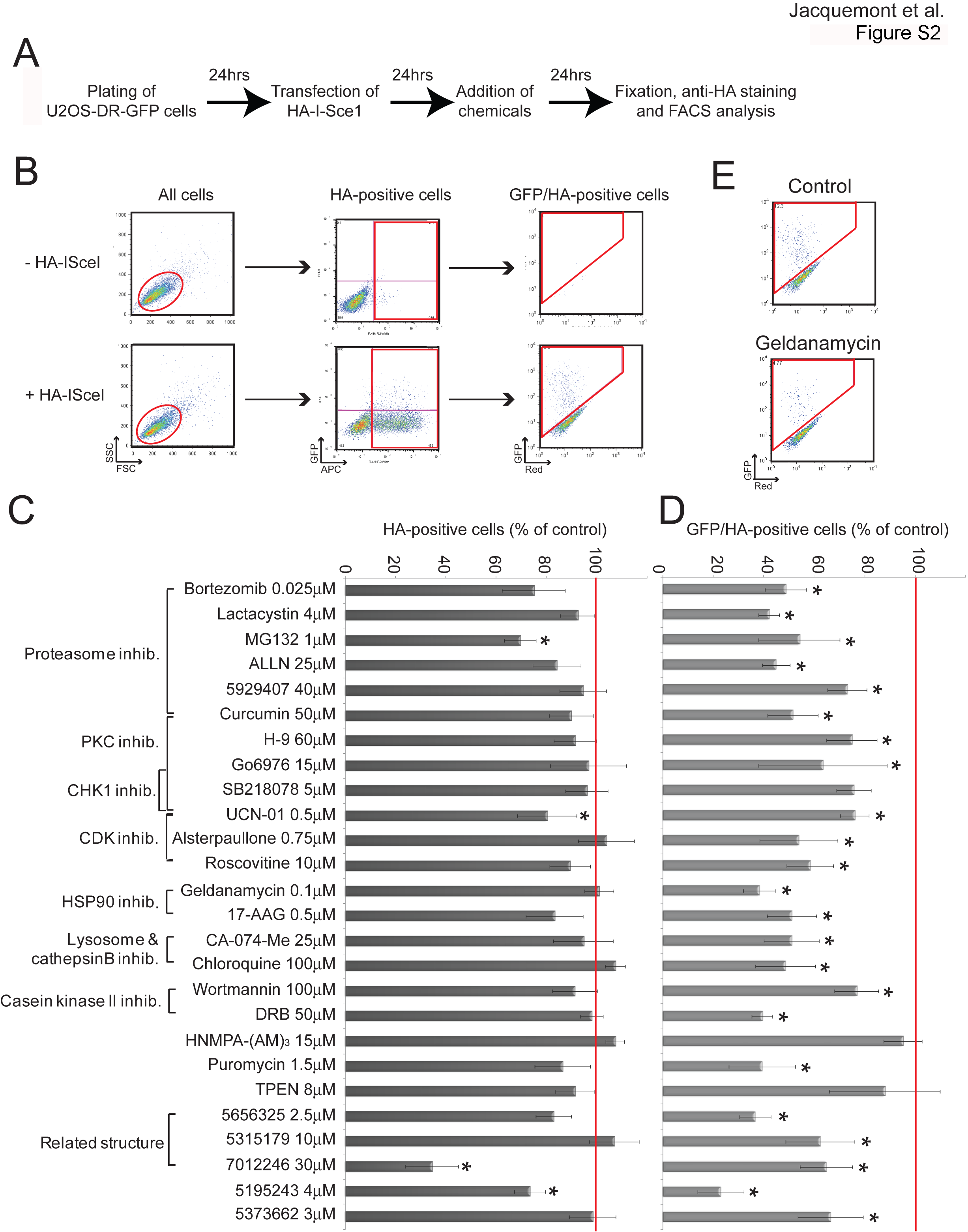

Supplement: Additional file 4 — Figure S2. Most FA pathway inhibitors inhibit HR. (A) Schematic of the HR assay performed using FA pathway inhibitors. (B) Flow cytometric analysis for HR efficiency using U2OS-DR-GFP cells and HA-tagged I-Sce1. Cells fixed and stained with anti-HA and APC-linked specific secondary antibodies were first gated in FCS/SSC scatter, then HA-positive population was gated in an APC/GFP dot plot and analyzed for GFP expression using a red/GFP dot plot. (C) The relative proportion of live cells compared to non-treated control cells in the HR assay in U2OS-DRGFP cells are shown (mean ± SEM (n=3 to 7)). Percentage of live cells was 95.0±1.2% in non-treated condition. (D) The relative proportion of cells expressing HA-tagged ISce1 compared to non-treated control cells in the HR assay in U2OS-DRGFP cells are shown (mean ± SEM (n=3 to 7)). An asterix (*) indicates significant decrease in proportion of HA-positive cells upon exposure to the indicated drug (p≤0.05, t test). Percentage of HA-positive cells was 39.2±2.5% in non-treated condition. (E) The relative proportion of GFP-positive cells in the HA-positive population, compared to non-treated control cells, is shown (mean ± SEM (n=3 to 7)). An asterix (*) indicates significant decrease in the proportion of GFP-positive/HA-positive cells upon exposure to the indicated drug (p≤0.05, t test). Percentage of GFP-positive/HA-positive cells was 9.5±0.9% in non-treated condition. These results are summarized in Figure 3. (F) Examples of flow cytometric profiles obtained with U2OS-DR-GFP cells untreated and treated with geldanamycin (0.1μM) for 24 hours. [file 1476-4598-11-26-S4.tiff]

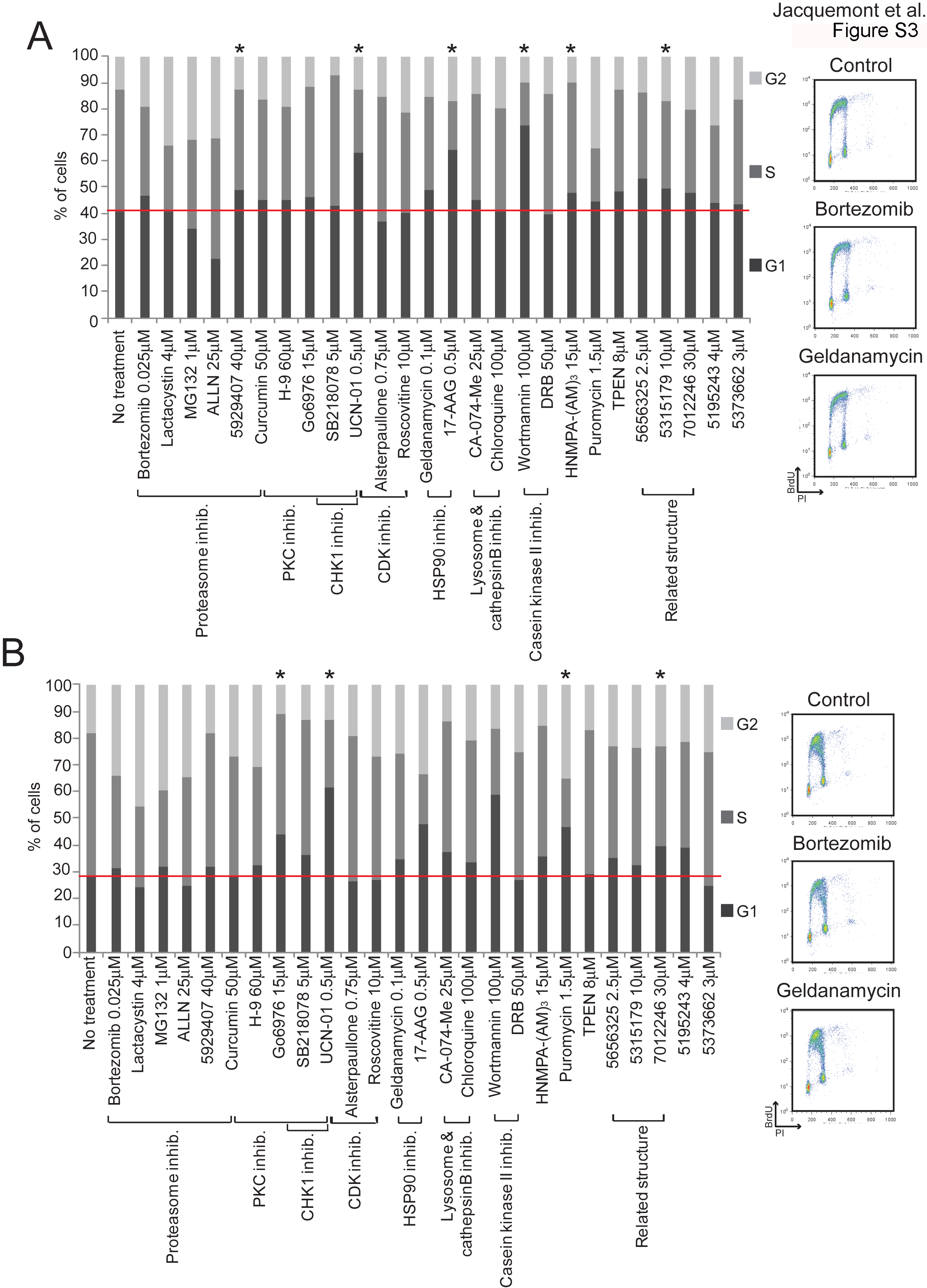

Supplement: Additional file 5 — Figure S3. Most FA pathway inhibitors do not significantly affect the proportion of cells in S+G2phases of the cell cycle. Cell cycle distribution quantified using BrdU/PI staining of U2OS-DR-GFP cells treated for 24 hours with the indicated drugs (A), or treated for 24 hours with the indicated drugs and irradiated with 10Gy 8 hours before the end of treatment (B) (n=3 to 4). An asterix (*) indicates significant decrease in proportion of S+G2 cells upon exposure to the indicated drug (p≤0.05, t test). Examples of flow cytometric profiles obtained with non-treated and FA pathway inhibitor-treated cells are shown. [file 1476-4598-11-26-S5.tiff]

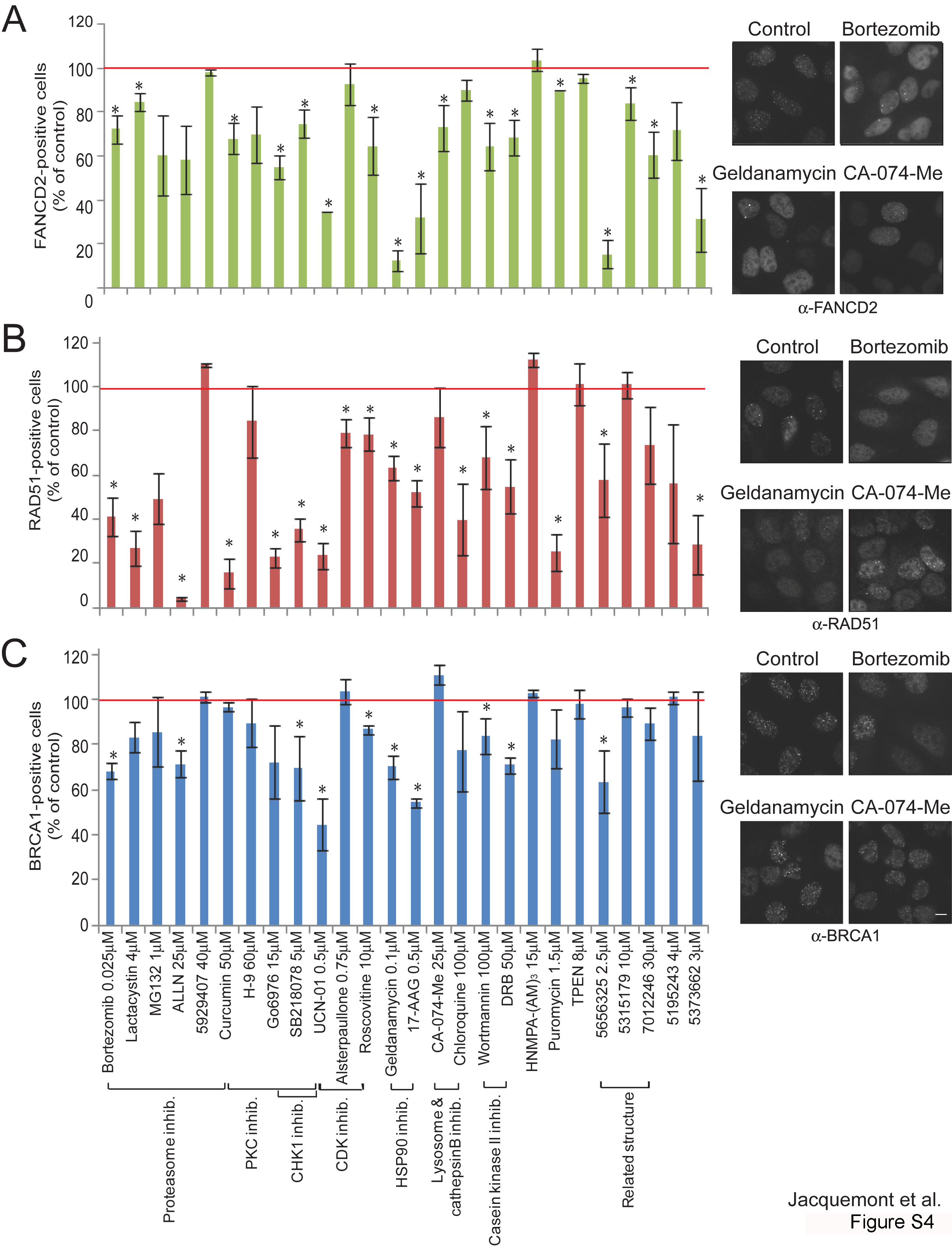

Supplement: Additional file 6 — Figure S4.Figure S2. Most FA pathway inhibitors inhibit HR. (A) Schematic of the HR assay performed using FA pathway inhibitors. (B) Flow cytometric analysis for HR efficiency using U2OS-DR-GFP cells and HA-tagged I-Sce1. Cells fixed and stained with anti-HA and APC-linked specific secondary antibodies were first gated in FCS/SSC scatter, then HA-positive population was gated in an APC/GFP dot plot and analyzed for GFP expression using a red/GFP dot plot. (C) The relative proportion of live cells compared to non-treated control cells in the HR assay in U2OS-DRGFP cells are shown (mean ± SEM (n=3 to 7)). Percentage of live cells was 95.0±1.2% in non-treated condition. (D) The relative proportion of cells expressing HA-tagged ISce1 compared to non-treated control cells in the HR assay in U2OS-DRGFP cells are shown (mean ± SEM (n=3 to 7)). An asterix (*) indicates significant decrease in proportion of HA-positive cells upon exposure to the indicated drug (p≤0.05, t test). Percentage of HA-positive cells was 39.2±2.5% in non-treated condition. (E) The relative proportion of GFP-positive cells in the HA-positive population, compared to non-treated control cells, is shown (mean ± SEM (n=3 to 7)). An asterix (*) indicates significant decrease in the proportion of GFP-positive/HA-positive cells upon exposure to the indicated drug (p≤0.05, t test). Percentage of GFP-positive/HA-positive cells was 9.5±0.9% in non-treated condition. These results are summarized in Figure 3. (F) Examples of flow cytometric profiles obtained with U2OS-DR-GFP cells untreated and treated with geldanamycin (0.1μM) for 24 hours. [file 1476-4598-11-26-S6.tiff]

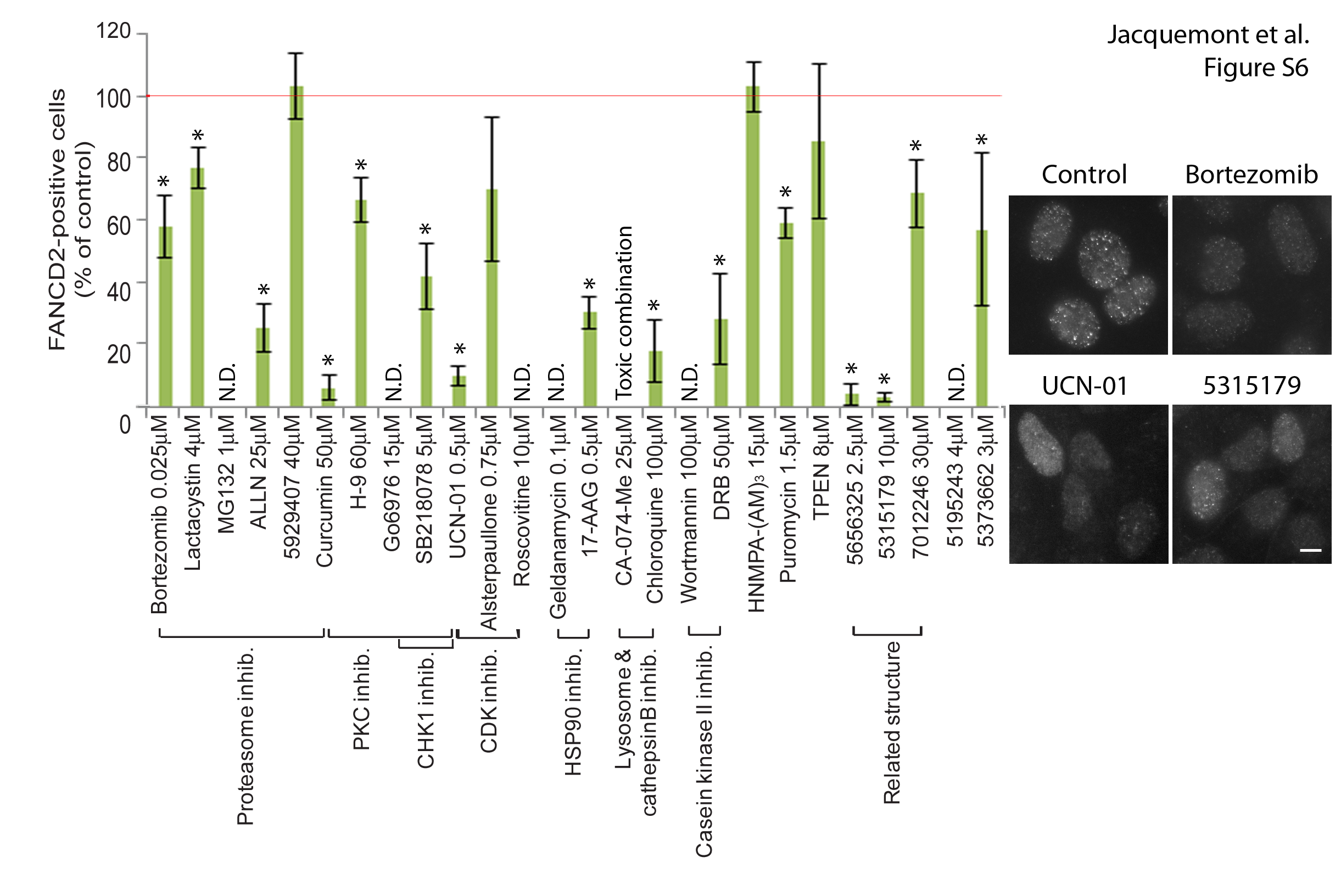

Supplement: Additional file 7 — Figure S6. Most FA pathway inhibitors significantly inhibit cisplatin-induced foci formation of FANCD2 in U2OS-DR-GFP cells. Relative proportion of U2OS-DR-GFP cells with more than 10 foci after 24hour incubation with 5μM cisplatin and the indicated drug, compared to controls (mean ± SEM (n=3)). An asterisk (*) indicates significant decrease in the proportion of foci-positive cells compared to controls (p ≤0.05, t test). In non-treated conditions, the percentage of FANCD2-positive cells was 83.13 ± 8.3%. Examples of immunofluorescence staining are also shown. Scale bar = 20μm. N.D. = not determined. [file 1476-4598-11-26-S7.tiff]

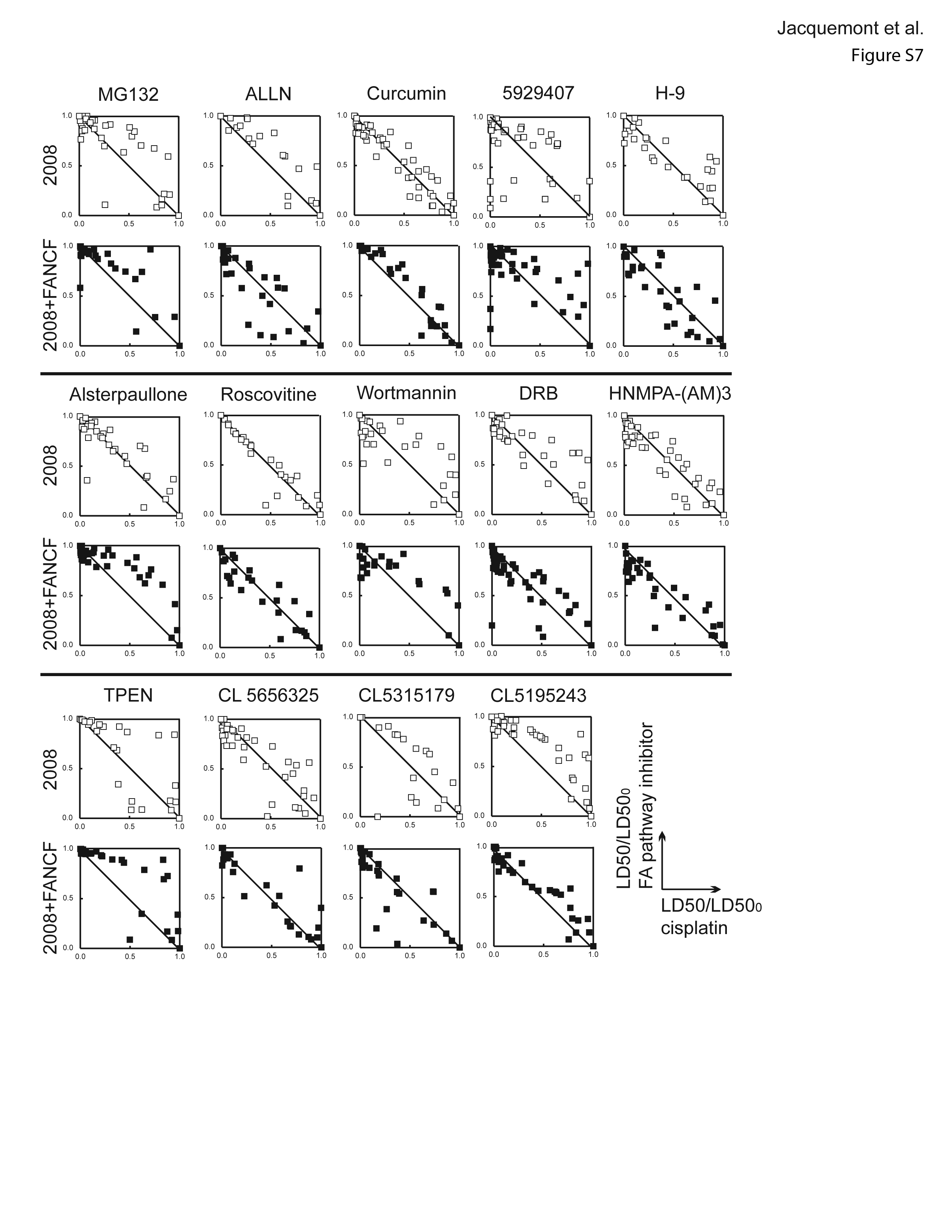

Supplement: Additional file 9 — Figure S7. Isobologram analyses at 50% killing of the FA pathway inhibitors that did not sensitize 2008 or 2008+FANCF ovarian cancer cells to cisplatin. Isobolograms at the LD50 level are presented, showing the results obtained in at least 3 independent experiments. Results are summarized in Table 2. [file 1476-4598-11-26-S9.tiff]

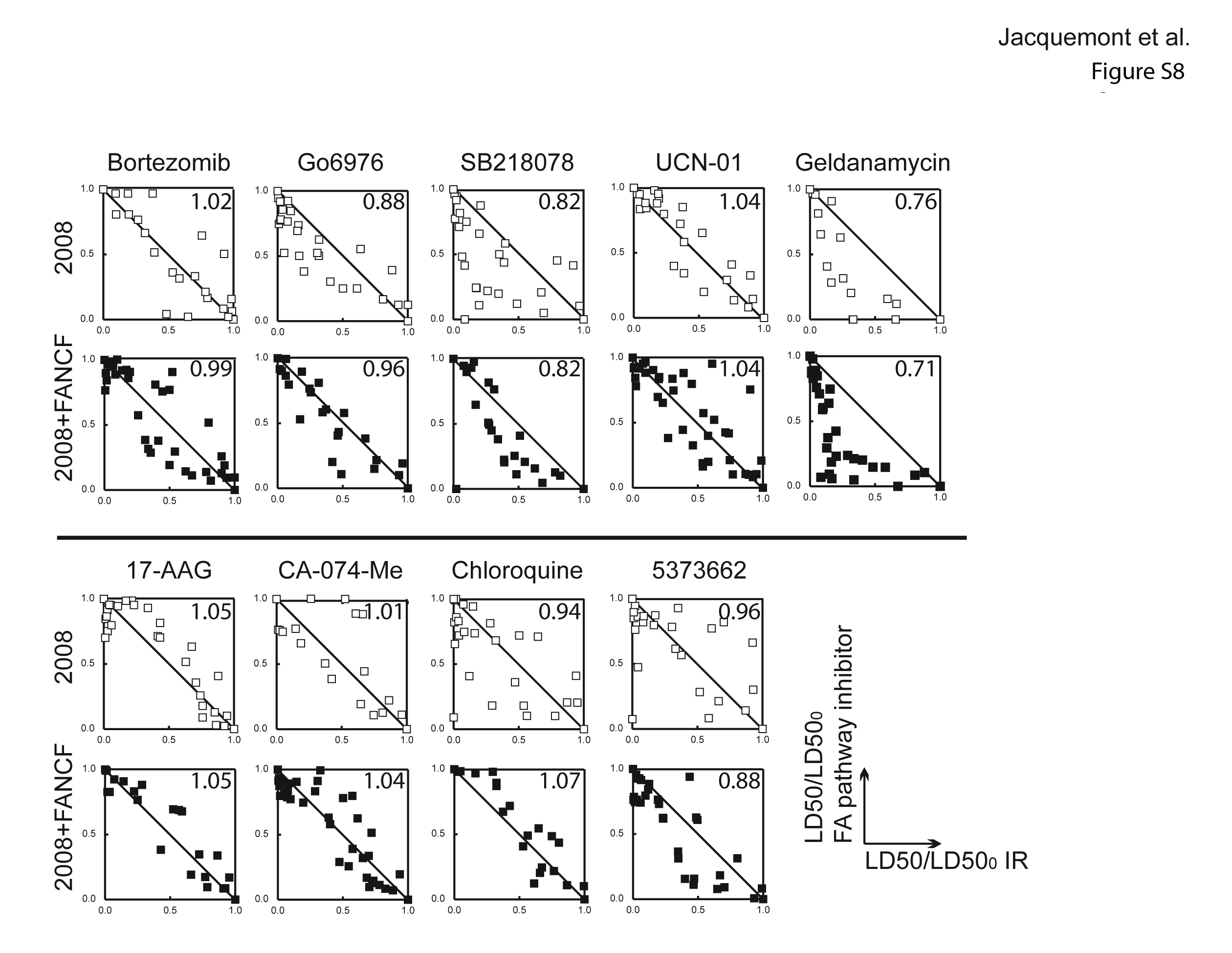

Supplement: Additional file 11 — Figure S8. Isobologram analyses for sensitization to IR of the FA pathway inhibitors that most significantly sensitize 2008 and/or 2008+FANCF ovarian cancer cells to cisplatin. Isobolograms at the LD50 level are presented, showing the results obtained in at least 3 independent experiments. Average combination index (CI) is indicated in the upper right corner of each isobologram: a CI≤0.90 indicates synergism. Results are summarized in Additional file 12: Table S4. [file 1476-4598-11-26-S11.tiff]
