# Supplementary material for: Non-specific chemical inhibition of the Fanconi anemia pathway sensitizes cancer cells to cisplatin
Source: Mol Cancer. 2012 Apr 26;11:26. doi: 10.1186/1476-4598-11-26 (PMC3478989; doi:10.1186/1476-4598-11-26)
Supplement: Additional file 8 — Table S2. 2008 and 2008+FANCF cells are equally sensitive to most FA pathway inhibitors. Lethal dose 50 (LD50) (mean ± SEM (n=3 to 6)) of the FA pathway inhibitors in 2008 and 2008+FANCF cells. An asterisk (*) indicates significant difference in sensitivity between 2008 and 2008+FANCF cells (p ≤0.05, paired t test). N.D. = not determined. Only two experiments were performed using lactacystin, because of the high concentration required to achieve 50% killing. [file 1476-4598-11-26-S8.docx]

|  | **Chemicals** |  | **LD50 (μM)** | | | | | | |
| --- | --- | --- | --- | --- | --- | --- | --- | --- | --- |
|  |  |  | **2008** | | |  | **2008+FANCF** | | |
|  |  |  | *FA-deficient* | | |  | *FA-proficient* | | |
|  | Bortezomib |  | 0.07 | ± | 0.01 |  | 0.10 | ± | 0.02 |
|  | Lactacystin |  | 40.80 | ± | N.D. |  | 36.66 | ± | N.D. |
|  | MG132 |  | 0.65 | ± | 0.04 |  | 0.57 | ± | 0.08 |
|  | ALLN |  | 13.05 | ± | 1.71 |  | 14.68 | ± | 2.13 |
|  | 5929407 |  | 31.60 | ± | 2.29 |  | 31.31 | ± | 2.06 |
|  | Curcumin |  | 19.53 | ± | 3.47 |  | 15.10 | ± | 0.92 |
|  | H-9 |  | 36.21 | ± | 4.82 |  | 32.64 | ± | 6.07 |
|  | Gö6976 |  | 0.85 | ± | 0.29 |  | 0.82 | ± | 0.27 |
|  | SB218078 |  | 3.53 | ± | 0.65 |  | 4.25 | ± | 0.73 |
|  | UCN-01 |  | 0.07 | ± | 0.01 |  | 0.10 | ± | 0.01 |
|  | Alsterpaullone |  | 1.80 | ± | 0.07 |  | 1.40 | ± | 0.04 |
|  | Roscovitine |  | 16.88 | ± | 0.60 |  | 16.58 | ± | 1.61 |
|  | Geldanamycin | * | **0.13** | **±** | **0.02** |  | **0.47** | **±** | **0.13** |
|  | 17-AAG |  | 0.08 | ± | 0.01 |  | 0.06 | ± | 0.01 |
|  | CA-074-Me |  | 7.45 | ± | 0.67 |  | 9.81 | ± | 1.20 |
|  | Chloroquine |  | 15.79 | ± | 2.23 |  | 9.98 | ± | 1.50 |
|  | Wortmannin |  | 47.08 | ± | 5.30 |  | 51.72 | ± | 7.67 |
|  | DRB |  | 43.39 | ± | 9.28 |  | 44.36 | ± | 5.53 |
|  | HNMPA-(AM)3 |  | 113.41 | ± | 18.45 |  | 116.73 | ± | 15.94 |
|  | Puromycin | * | **0.59** | **±** | **0.06** |  | **2.77** | **±** | **0.31** |
|  | TPEN |  | 7.31 | ± | 0.14 |  | 7.46 | ± | 0.18 |
|  | 5656325 |  | 0.98 | ± | 0.07 |  | 0.97 | ± | 0.08 |
|  | 5315179 |  | 3.22 | ± | 0.35 |  | 3.96 | ± | 0.34 |
|  | 7012246 |  | 16.66 | ± | 0.74 |  | 20.31 | ± | 2.86 |
|  | 5195243 |  | 2.06 | ± | 0.11 |  | 2.26 | ± | 0.05 |
|  | 5373662 |  | 1.74 | ± | 0.32 |  | 3.07 | ± | 1.41 |
|  | Cisplatin | * | **0.67** | **±** | **0.04** |  | **3.52** | **±** | **0.28** |
|  | IR | * | **4.28** | **±** | **0.18(Gy)** | | **5.24** | **±** | **0.17(Gy)** |

**Table S2. 2008 and 2008+FANCF cells are equally sensitive to most FA pathway inhibitors.**

Lethal dose 50 (LD50) (mean ± SEM (n=3 to 6)) of the FA pathway inhibitors in 2008 and 2008+FANCF cells. An asterisk (*) indicates significant difference in sensitivity between 2008 and 2008+FANCF cells (p ≤0.05, paired t test). N.D. = not determined. Only two experiments were performed using lactacystin, because of the high concentration required to achieve 50% killing.
