# Supplementary material for: Non-specific chemical inhibition of the Fanconi anemia pathway sensitizes cancer cells to cisplatin
Source: Mol Cancer. 2012 Apr 26;11:26. doi: 10.1186/1476-4598-11-26 (PMC3478989; doi:10.1186/1476-4598-11-26)
Supplement: Additional file 10 — Table S3. Drug interactions at 70% killing between cisplatin and the FA pathway inhibitors in FA pathway-deficient and -proficient ovarian cancer cells. Combination index (CI) at 70% killing values (mean ± SEM) calculated from isobologram at the LD70 level analyses of combination of cisplatin with all FA pathway inhibitor, performed in an FA-deficient (2008) and an FA-proficient (2008+FANCF) ovarian cancer cell lines. Synergism is indicated in bold text. [file 1476-4598-11-26-S10.docx]

|  | | | | | | | | | | | |
| --- | --- | --- | --- | --- | --- | --- | --- | --- | --- | --- | --- |
|  | | | **Interaction with cisplatin at 70% killing** | | | | | | | | |
|  |  |  | **2008** | | | |  | **2008+FANCF** | | | |
| **Chemicals** | | | *FA-deficient* | | | |  | *FA-proficient* | | | |
|  |  |  | **CI** | | | **Interpretation** |  | **CI** | | | **Interpretation** |
|  | Bortezomib | | 0.86 | ± | 0.03 | **Slight synergism** |  | 0.81 | ± | 0.04 | **Moderate synergism** |
|  | Lactacystin | | N.D. | | | - |  | N.D. | | | - |
|  | MG132 |  | 1.06 | ± | 0.06 | Additive |  | 1.10 | ± | 0.06 | Additive |
|  | ALLN |  | 1.07 | ± | 0.03 | Additive |  | 0.85 | ± | 0.06 | **Moderate synergism** |
|  | 5929407 |  | 1.10 | ± | 0.03 | Additive |  | 1.14 | ± | 0.05 | *Slight antagonism* |
|  | Curcumin |  | 0.98 | ± | 0.02 | Additive |  | 0.99 | ± | 0.03 | Additive |
|  | H-9 |  | 0.98 | ± | 0.04 | Additive |  | 0.91 | ± | 0.03 | Additive |
|  | Gö6976 |  | 0.56 | ± | 0.03 | **Synergism** |  | 0.58 | ± | 0.07 | **Synergism** |
|  | SB218078 | | 0.97 | ± | 0.06 | Additive |  | 0.85 | ± | 0.03 | **Moderate synergism** |
|  | UCN-01 |  | 0.78 | ± | 0.03 | **Moderate synergism** |  | 0.58 | ± | 0.04 | **Synergism** |
|  | Alsterpaullone | | 0.96 | ± | 0.02 | Additive |  | 1.09 | ± | 0.05 | Additive |
|  | Roscovitine | | 0.98 | ± | 0.02 | Additive |  | 0.97 | ± | 0.02 | Additive |
|  | Geldanamycin | | 0.63 | ± | 0.07 | **Synergism** |  | 0.29 | ± | 0.10 | **Strong synergism** |
|  | 17-AAG |  | 0.83 | ± | 0.02 | **Moderate synergism** |  | 0.74 | ± | 0.02 | **Moderate synergism** |
|  | CA-074-Me | | 0.77 | ± | 0.03 | **Moderate synergism** |  | 0.73 | ± | 0.02 | **Moderate synergism** |
|  | Chloroquine | | 0.84 | ± | 0.04 | **Moderate synergism** |  | N.D | | | - |
|  | Wortmannin | | 1.05 | ± | 0.05 | Additive |  | 1.04 | ± | 0.04 | Additive |
|  | DRB |  | 0.97 | ± | 0.02 | Additive |  | 0.94 | ± | 0.02 | Additive |
|  | HNMPA-(AM)3 | | 0.94 | ± | 0.05 | Additive |  | 0.93 | ± | 0.02 | Additive |
|  | TPEN |  | 1.16 | ± | 0.05 | *Slight antagonism* |  | 1.18 | ± | 0.05 | *Slight antagonism* |
|  | 5656325 |  | 0.97 | ± | 0.02 | Additive |  | 0.89 | ± | 0.03 | **Slight synergism** |
|  | 5315179 |  | 1.11 | ± | 0.05 | *Slight antagonism* |  | 0.89 | ± | 0.06 | **Slight synergism** |
|  | 7012246 |  | 1.19 | ± | 0.05 | *Slight antagonism* |  | 1.06 | ± | 0.05 | Additive |
|  | 5195243 |  | 1.12 | ± | 0.03 | *Slight antagonism* |  | 0.94 | ± | 0.03 | Additive |
|  | 5373662 |  | 0.98 | ± | 0.03 | Additive |  | 0.83 | ± | 0.05 | **Moderate synergism** |
|  |  |  |  |  |  |  |  |  |  |  |  |

**Table S3. Drug interactions at 70% killing between cisplatin and the FA pathway inhibitors in FA pathway-deficient and -proficient ovarian cancer cells.**

Combination index (CI) at 70% killing values (mean ± SEM) calculated from isobologram at the LD70 level analyses of combination of cisplatin with all FA pathway inhibitor, performed in an FA-deficient (2008) and an FA-proficient (2008+FANCF) ovarian cancer cell lines. Synergism is indicated in bold text.
