# Supplementary material for: Non-specific chemical inhibition of the Fanconi anemia pathway sensitizes cancer cells to cisplatin
Source: Mol Cancer. 2012 Apr 26;11:26. doi: 10.1186/1476-4598-11-26 (PMC3478989; doi:10.1186/1476-4598-11-26)
Supplement: Additional file 12 — Table S4. Interactions at 50% killing between IR and the FA pathway inhibitors in FA pathway-deficient and -proficient ovarian cancer cells. Combination index (CI) at 50% killing values (mean ± SEM) calculated from isobologram at the LD50 level analyses of combination of IR with each FA pathway inhibitor, performed in FA-deficient (2008) and FA-proficient (2008+FANCF) ovarian cancer cell lines (see Additional file 11: Figure S8). Synergism is indicated in bold text. [file 1476-4598-11-26-S12.docx]

|  |  |  |  |  |  |  |  |  |  |  |  |
| --- | --- | --- | --- | --- | --- | --- | --- | --- | --- | --- | --- |
|  | | | **Interaction with IR at 50% killing** | | | | | | | | |
|  |  |  | **2008** | | | |  | **2008+FANCF** | | | |
| **Chemicals** | | | *FA-deficient* | | | |  | *FA-proficient* | | | |
|  |  |  | **CI** | | | **Interpretation** |  | **CI** | | | **Interpretation** |
|  | Bortezomib | | 1.02 | ± | 0.05 | Additive |  | 0.99 | ± | 0.03 | Additive |
|  | Gö6976 |  | 0.88 | ± | 0.03 | **Slight synergism** |  | 0.96 | ± | 0.03 | Additive |
|  | SB218078 | | 0.82 | ± | 0.06 | **Moderate synergism** |  | 0.82 | ± | 0.06 | **Moderate synergism** |
|  | UCN-01 |  | 1.04 | ± | 0.03 | Additive |  | 1.04 | ± | 0.04 | Additive |
|  | Geldanamycin | | 0.76 | ± | 0.06 | **Moderate synergism** |  | 0.71 | ± | 0.06 | **Moderate synergism** |
|  | 17-AAG |  | 1.05 | ± | 0.04 | Additive |  | 1.05 | ± | 0.03 | Additive |
|  | CA-074-Me | | 1.01 | ± | 0.06 | Additive |  | 1.04 | ± | 0.04 | Additive |
|  | Chloroquine | | 0.94 | ± | 0.05 | Additive |  | 1.07 | ± | 0.03 | Additive |
|  | 5373662 |  | 0.96 | ± | 0.07 | Additive |  | 0.88 | ± | 0.03 | **Slight synergism** |

**Table S4. Interactions at 50% killing between IR and the FA pathway inhibitors in FA pathway-deficient and -proficient ovarian cancer cells.**

Combination index (CI) at 50% killing values (mean ± SEM) calculated from isobologram at the LD50 level analyses of combination of IR with each FA pathway inhibitor, performed in FA-deficient (2008) and FA-proficient (2008+FANCF) ovarian cancer cell lines (see Figure S8). Synergism is indicated in bold text.
